# Modulation of Oral Bioavailability and Metabolism for Closely Related Cyclic Hexapeptides

**DOI:** 10.1007/s10989-017-9590-8

**Published:** 2017-03-28

**Authors:** Thomas Vorherr, Ian Lewis, Joerg Berghausen, Sandrine Desrayaud, Michael Schaefer

**Affiliations:** Novartis Institutes for Biomedical Research, 4002 Basel, Switzerland

**Keywords:** Conformation, Oral bioavailability, Peptide metabolism, Permeability, Pharmacokinetics

## Abstract

**Abstract:**

Recently, a variety of studies concerned with the permeability and oral bioavailability of cyclic peptides have been reported. In particular, strategies aiming at modifying peptides to maintain or to enhance solubility while enabling permeability constitute a significant challenge, but are of high interest to ensure a smooth drug discovery process. Current methodologies include *N*-methylation, matching of hydrogen bonding acceptors and donors across the macrocycle, and additional masking of polarity. In this study, we investigate further the pivotal effects of shielding on permeability and studied the metabolism of the corresponding peptides in more detail by comparing peptide concentrations in the portal versus the jugular vein in rats. Interestingly, minor changes in one particular side chain impacts both permeability and liver metabolism.

**Graphical Abstract:**

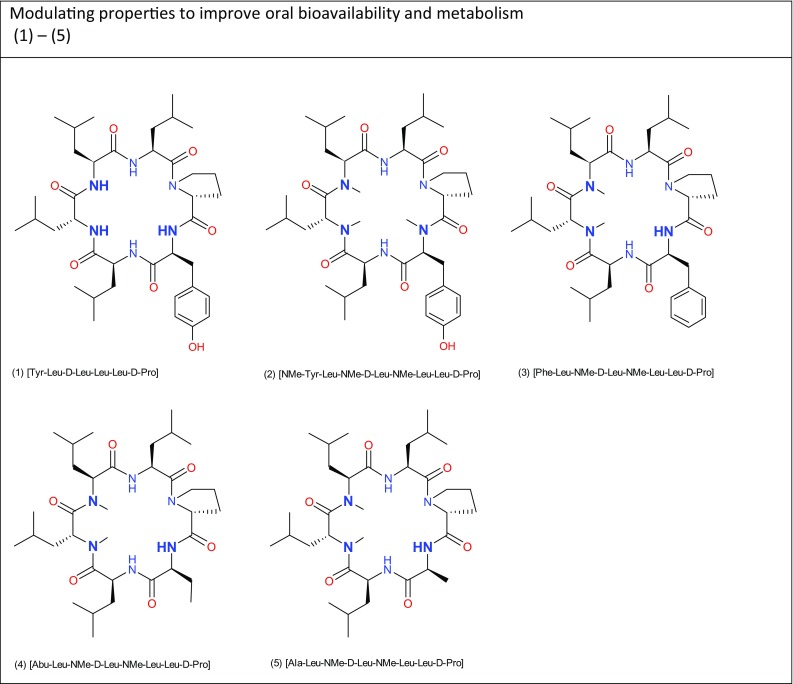

**Electronic supplementary material:**

The online version of this article (doi:10.1007/s10989-017-9590-8) contains supplementary material, which is available to authorized users.

## Introduction

The improvement of absorption, distribution, metabolism, and excretion (ADME) properties for peptides is of utmost importance to broaden applications for macromolecules, e.g. to enable delivery by the oral route. Principal studies to elucidate the role of the backbone and side chains for a given cyclic peptide with respect to permeability, and in particular oral uptake, are of value to learn more about the required property space. In principle, this know-how can then be systematically applied to design permeable and potent macromolecules in an iterative fashion, and should encourage a straight forward development of orally bioavailable peptide therapeutics. Especially, the promotion of permeability while maintaining a good solubility remains a significant challenge and is of interest in our investigation (Marzinzik and Vorherr 2013; Vorherr 2015; Kwon and Kodadek 2007). This intense effort on macromolecules has become extremely important, since for many attractive drug targets no high affinity and selective small molecules complying with the “rule-of-five” (Krämer et al. 2016; Whitty et al. 2016) can be identified. This rule, originally published by Lipinski et al. (2001), represents an empirical evaluation of the suitability of a given molecule or class of molecules towards favorable oral bioavailability in humans. However, macromolecules were not in the focus, and the original guidelines, e.g. molecular weight ≤ 500, were extended (molecular weight ≤ 720 and polar surface area remaining at ≤140 Å^2^) (Veber et al. 2002). More recently (Villar et al. 2014), a molecular weight range of 600–1200 and values for the polar surface area of 180–320 Å^2^ were reported as a potential space for oral drugs. Thus, stepping outside the limited chemical space seems possible and may offer opportunities to find molecules with high potency and selectivity. However, there is a need to obtain appropriate properties to render particular ligands druggable even though the route of administration requires transport across certain barriers, e.g. crossing the intestinal mucosa to achieve systemic exposure.

**Fig. 1:**
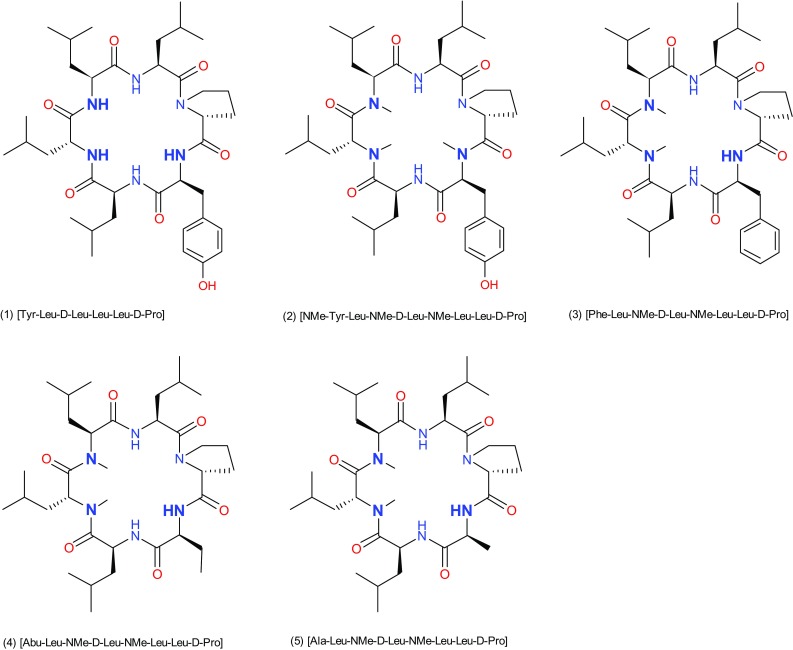
Cyclohexapeptides (1)–(5)

In a previous investigation, we introduced the concept of a polar surface assessment in 3D called solvent-accessible polar surface area (SAPSA), and showed the correlation of in vitro permeability on cyclic hexapeptides (1) and (2) (Fig. 1) with the in vivo oral bioavailability (%BAV) as indicated in Table 2 for peptides (1) and (2) (Lewis et al. 2015). However, taking peptide (2) as an example of multiple *N*-methylation, solubility is considerably reduced, and as expected, a substantial increase of oral bioavailability was observed. In a recent publication, effects of *N*-methylation on aqueous solubility based on considerations of increased polarity and flexibility have been suggested from an *in-silico* assessment of the various amino acids and their corresponding *N*-methylated counterparts (Rauf et al. 2015). In these calculations, all *N*-methylated amino acid derivatives showed a larger dipole moment as compared to the non-methylated amide bond. Since the contribution of single dipoles to polarity in the context of a cyclic peptide is not clear to us, in this contribution, we define polarity as the ability to interact with H-bond acceptors and donors. In order to ensure an optimal development path, we thought of modifications improving solubility while maintaining permeability. Furthermore, we wanted to study structurally related derivatives to enable comparability with the previous studies. In a first step, we decided to reduce the degree of *N*-methylation. Indeed, a variety of publications on *N*-Methylation have emerged (Krämer et al. 2016; Biron et al. 2008; White et al. 2011). Most interestingly, a recent design to improve on the natural product Sanguinamide A (Nielsen et al. 2012) clearly showed that *N*-methylation does not always enhance oral uptake (Nielsen et al. 2014). Of course, the question may arise whether a lower degree of *N*-methylation encourages flexibility in a cyclic peptide (Rauf et al. 2015), and as a consequence, adds more or less chameleonic properties to the molecule (Whitty et al. 2016). More generally, we aimed to increase the understanding of how to modulate properties of macrocyclic drugs (Giordanetto and Kihlberg 2014), and to rationalize the impact of structural features enhancing “drug-like” properties (Over et al. 2014). Special building blocks enabling shielding or masking of polarity by formation of intramolecular hydrogen bonds have been reported (Rafi et al. 2012), however in the context of these studies focused on peptides (3)–(5), we concentrated on replacing the polar Tyr to study the influence of differentially sized apolar groups. In addition to our particular 3D polar surface calculation called SAPSA and the more classical profiling by parallel artificial membrane permeability assay (PAMPA) and Madin Darby canine kidney cells (MDCK) applied earlier (Lewis et al. 2015), we used the experimental determination of polar surfaces as measured by supercritical fluid chromatography (SFC) (Goetz et al. 2014a, b) to characterize cyclic peptides for permeability. The focus of this study was to better understand contributions of intestinal uptake and clearance/metabolism to the observed exposure upon oral administration in the selected model systems. In our opinion, a deeper understanding of certain pharmacokinetic parameters will provide guidance how to improve properties, and as a consequence, how oral bioavailability is influenced from this perspective.

## Methods

### Synthesis

Amino acids, reagents and solvents were purchased from Bachem or Aldrich. Two synthetic strategies were utilized in the preparation of the cyclic peptides described. Cyclic peptides (1) and (2) were synthesized, using the first synthetic strategy, starting with the allyl ester of fluorenylmethyloxycarbonyl (Fmoc)-protected tyrosine linked to a trityl polystyrene resin (TentaGel S Trt Cl from RAPP Polymere GmbH) via the –OH of the tyrosine side-chain. Cyclization was performed on-resin, after deprotection of the allyl ester, followed by cleavage of the cyclic peptide from the resin, as previously described (Lewis et al. 2015). Peptides (3)–(5) were prepared utilizing the second synthetic strategy, with attachment of the Fmoc-D-Pro residue via the C-terminal carboxy group to the 2-Cl-Trt-resin and assembly of the linear peptide on the resin. Peptides were prepared using a Symphony automated peptide synthesizer, cleaved from the resin, subsequently cyclized in solution and purified by prep. HPLC. Typically, peptides were synthesized as follows: Fmoc removal was performed using 4-methylpiperidine/DMF 1:4 (2 × 5 min, 5 mL). The resin was washed with DMF (×3) and DCM (×4) for 10 min each. Coupling of AAs was performed using 2 Eq. of Fmoc-AA-OH, 4.0 Eq. of HATU and 8.0 Eq. of DIPEA at room temperature. Subsequently, the resin was washed with DMF (×3) and DCM (×4). The linear peptide resin was cleaved from the resin by shaking at room temperature for 3 × 10 min then 1 × 5 min with HFIP/DCM (25:75) (6 mL each time). The resin was then washed with DCM (2×) and the cleavage and washing solutions were filtered off then concentrated to dryness in vacuo. Subsequent to cleavage, cyclization was carried out in solution. Typically a 500 mL round-bottomed flask was equipped with a magnetic stirrer and stirring was carried out at 40 °C for several hours. Preparative reversed phase purification was carried out as follows: Column: Waters XBridge Prep C18 5 μm OBD, 30 × 250 mm; Waters Part No 186004025; Eluent A: 0.1% TFA in water; Eluent B: MeCN; Flow: 30 mL/min; Gradient : 30%B in 30 min 100%B, fractions collected with a Waters 2767 Sample Manager and UV detection with a Waters 2996 Photo Diode Array Detector linked to a Waters 2525 Binary Gradient Module Pump.

Compound (3): H_2_N-Phe-Leu-NMe-D-Leu-NMe-Leu-Leu-D-Pro-OH (216 mg, 0.291 mmol) was dissolved in DCM (90 mL) to provide solution A. Solution B was prepared by dissolving HOAt (59.4 mg, 0.436 mmol), HATU (442 mg, 1.163 mmol) and 2,6-lutidine (1.016 mL, 8.72 mmol) in DCM (200 mL). Then solution A was added to solution B dropwise and the reaction was stirred at RT for 2 h then concentrated to dryness in vacuo. Purification was carried out by preparative HPLC eluting from 5%MeCN/water to 70%MeCN/water in 17 min and pure fractions were combined and lyophilized, affording the desired compound (3) [Phe-Leu-NMe-D-Leu-NMe-Leu-Leu-D-Pro] (110 mg, 0.152 mmol, 52.2% yield, >98% purity, (M + H) + = 725.49) as a white lyophilisate.

Compound (4): H_2_N-Abu-Leu-NMe-D-Leu-NMe-Leu-Leu-D-Pro-OH (136 mg, 0.200 mmol), was dissolved in DCM (50 mL) to provide solution A. Solution B was prepared by dissolving HOAt (40.8 mg, 0.300 mmol) and HATU (304 mg, 0.800 mmol) in DCM (200 mL) and 2,6-lutidine (0.699 mL, 6.00 mmol). Then solution A was added to solution B dropwise and the reaction was stirred at 40 °C for 2h, then concentrated to dryness in vacuo. Purification was carried out by preparative HPLC eluting from 5%MeCN/water to 70%MeCN/water in 17 min and pure fractions were combined and lyophilized, affording the desired compound (4) [Abu-Leu-NMe-D-Leu-NMe-Leu-Leu-D-Pro] (47.8 mg, 0.072 mmol, 36.1% yield, >98% purity, (M + H) + = 663.48) as a white lyophilisate.

Compound (5): H_2_N-Ala-Leu-NMe-D-Leu-NMe-Leu-Leu-D-Pro-OH (300 mg, 0.450 mmol) was dissolved in MeCN (35 mL) to provide solution A. Solution B was prepared by dissolving HATU (513 mg, 1.350 mmol, 3 eq.) and DIPEA (470 μL, 2.70 mmol, 6.0 eq.) in MeCN (25 mL). Then, solution 1 was added dropwise (90 mL/h) in 20 min to solution 2 and the reaction was stirred for 1 h at RT, then concentrated to dryness in vacuo. Purification was carried out by preparative HPLC eluting from 5%MeCN/water to 70%MeCN/water in 17 min and pure fractions were combined and lyophilized, affording the desired compound (5) [Ala-Leu-NMe-D-Leu-NMe-Leu-Leu-D-Pro] (120 mg, 0.178 mmol, 39.5% yield, >95% purity, (M + H) + = 649.5) as a white lyophilisate.

Analytical data (UPLC, MS, NMR) is contained in the supplementary information including the chromatograms and spectra in Figures S1 and S2.

### Assays for Microsomal Clearance, Permeability, Protein Binding, and Solubility

Protocols for high-throughput equilibrium solubility, PAMPA, cellular permeability by the native MDCK assay, plasma protein binding, and microsomal clearance experiments were followed as reported earlier (Lewis et al. 2015). The values relate to single experiments. For the MDCK assay, the lower limit of quantification (LLOQ) was 5 nM, and for PAMPA 2 nM, respectively. For both PAMPA and MDCK, the standard deviation in log units for those assays is 0.2 (1.6-fold on a linear scale). This has been estimated by looking at independent replicates across the assay dynamic range.

### SAPSA

Molecular dynamics simulations were performed over 20 ns for every peptide using an implicit solvent model of water (Schaefer and Karplus 1996) generating a set of 20,000 conformations for each as previously reported (Lewis et al. 2015). The initial 3-dimensional structures were generated using the program Corina (Sadowski et al. 1994). Before starting data collection, the peptides were minimized for 400 steps and heated to 300 K by performing 10 ps Langevin dynamics. A water model was applied for the dynamics, rather than a model representing the membrane interior (e.g., chloroform), since this leads to faster transitions between conformations in the simulations and better sampling of conformation space (Chen et al. 2008). For every structure thus obtained, we computed the 3-dimensional solvent-accessible polar surface area. By averaging over all conformations, we determined the time-averaged, solvent accessible surface area (SAPSA) of the peptides. Conformational changes and the dynamic formation of hydrogen bonds were facilitated by a high dielectric constant of 80 representing water as the solvent, and a low friction constant of 1/ps for performing constant temperature Langevin dynamics (Brooks et al. 1983). The SAPSA for each frame is determined as the area covered by the center of a solvent probe sphere with a radius of 1.4 Å in contact with oxygen, nitrogen, and their bonded hydrogens (Richmond 1984). For the solute atoms, we employ the same Bondi radii as are used to compute the conventional polar surface area (PSA) (Ertl et al. 2000). A more detailed comparison was carried out for the two closely related peptides (4) and (5). In order to verify, whether a reduced SAPSA is the result of direct shielding or is induced by the side-chain affecting the conformation of the scaffold, in the trajectory of peptide (4), the terminal CH_3_-group of Abu was replaced by a hydrogen atom to match the structure of peptide (5). Then, the SAPSA was calculated for this artificial trajectory and compared to the outcome from the standard simulations of peptides (4) and (5) (Fig. 3).

### SFC

Conditions for analytical supercritical fluid chromatography (SFC) were taken from previous publications (Goetz et al. 2014a, b). The Pirkle chiral stationary phase Chirex 3014, a silica bonded (S)-valine and (R)-1-(α-naphthyl)ethylamine with a urea linkage, was selected. A mobile phase of 15% methanol in supercritical CO_2_ enabled adequate separation in combination with a low-slope gradient. High retention times were mitigated by increasing the percentage of methanol in the eluent and the addition of the relatively weak salt pair ammonium formate. In the resulting chromatographic system, differences in retention depend upon hydrophobic interactions, the ability for hydrogen bonding, the presence of dipoles, and van der Waals interactions of the solutes with the stationary phase (Goetz et al. 2014a, b).

### Proteolytic Stability

Peptides (1)–(5) were studied for stability in rat blood, kidney, and liver homogenate. Pre-weighed tissues from rat were homogenized with three part volume of DPBS buffer by the Dispomix™ device. 5 μL of the corresponding working solution (c = 400 μM in dimethylsulfoxide) were spiked in 400 μL pre-warmed to 37 °C matrix. At the time points 0, 0.25, 0.5, 1, 2 and 4 h (in duplicate), an aliquot of 30 μL was taken and added to 200 μL MeCN (including Glyburide c = 50 ng/mL as an internal standard for protein precipitation). Sample analysis was performed on a Thermo Finnigan Q Exactive hybrid quadrupole-Orbitrap mass spectrometer equipped with a Heated Electrospray Ionization (HESI-II) Probe (Waltham, Massachusetts, U.S.A.). The MS system was connected to a Thermo Scientific Dionex UltiMate 3000 System (Waltham, Massachusetts, U.S.A.). The supernatants (2 µL) were injected directly onto the LC-HRMS system for analysis. The test articles and its internal standard were separated with a Phenomenex Kinetex C18 (50 × 2 mm ID, 2.7 µm pore size). A binary gradient with a mobile phase consisting of water (A) and MeCN (B) was used for the LC-separation. The both mobile phases (A) and (B) were acidified with 0.1% formic acid. The elution gradient program was as follows: [time (min), (% mobile phase B): (0, 30) (4, 90) (4.1, 98) (5, 98) (5.1, 30) (7, 30)]. The column temperature was maintained at 50 ^°^C using a column heater. Under these experimental conditions, the LLOQ was 10 ng/mL.

### In Vivo Studies

Mouse PK studies on peptides (1)–(3) were performed in OF1 mice at a dosing of 2.1 mg/kg i.v. and 7.1 mg/kg p.o. [peptides (1) and (2)], and 1 mg/kg i.v. and 7.5 mg/kg p.o. for peptide (3) as described earlier (Lewis et al. 2015). For mouse PK on peptides (4) and (5), blood concentrations versus time profiles were obtained from 2 groups of 3 male C57Bl/6 mice. In the intravenous PK group (n = 3), the compound was administered intravenous (i.v.) by bolus injection (5 mL/kg) at a dose of 1 mg/kg, solubilized in *N*-Methyl-2-pyrrolidone (10%) and blank plasma (90%). For oral PK, another group of 3 male C57Bl/6 mice, a dose of 3 mg/kg was orally applied (dosing volume of 10 mL/kg) as a homogenous suspension of water (99%), Tween80 (0.5%) and methylcellulose (0.5%).

For rat PK, blood concentration versus time profiles were obtained from 3 male Sprague Dawley rats. For intravenous PK, the compound was administered intravenously by bolus injection (0.5 mL/kg) at a dose of 3 mg/kg, solubilized in *N*-Methyl-2-pyrrolidone (30%) and PEG200 (70%). For oral PK, after a washout period of 48 h, an oral gavage was administered to the same animals at a dose of 3 mg/kg at a dosing volume of 2.5 mL/kg. The oral dose was dispersed in water (99%), Tween80 (0.5%) and methylcellulose (0.5%), forming a homogenous suspension. For both rodents, blood samples (10–50 µL) were collected at 0.08 (i.v. only), 0.25, 0.5, 1, 2, 4, 7, and 24 h after dosing. To investigate the absorption and the impact of pre-systemic metabolism, the model of portal vein cannulation in rats was applied for compounds orally dosed in a cassette. The week before the PK experiments, the rats were surgically prepared with catheter implantation into the right jugular vein (for collection of blood from the systemic circulation) and the hepatic portal vein (for collection of blood from the portal vein, i.e. after intestinal absorption and metabolism and before liver pe-systemic metabolism). After recovery, the rats were orally dosed by gavage (same dose, volume and formulation as described above), and blood samples (50 µL) were collected at 0.25, 0.5, 1, 2, 4, 7, and 24 h concomitantly from the 2 blood vessels. Analyses of parent compound concentrations were carried out in blood using LC-MS/MS. An aliquot of 30 μL was taken and added to 200 μL MeCN (including Glyburide (c = 50 ng/mL) as internal standard) for protein precipitation. Sample analysis was performed on a LC–MS/MS system consisting of an AB SCIEX API 5500 QTrap mass spectrometer equipped with a TurboIonSpray™ interface (Framingham, USA). The MS system was connected to a HTS CTC PAL auto-sampler (Zwingen, Switzerland) and to a Flux Rheos 2200 pump system (Reinach, Switzerland). The supernatants (2 µL) were injected directly onto the LC-MS/MS system for analysis. The test articles and its internal standard were separated with a Phenomenex Kinetex C18 (50 × 2 mm ID, 2.7 µm pore size). A binary gradient with a mobile phase consisting of water (A) and acetonitrile (B) was used for the LC-separation. Both mobile phases (A) and (B) were acidified with 0.1% formic acid. The elution gradient program was as follows: [time (min), (% mobile phase B): (0, 95) (5.8, 85) (5.81, 99) (6.5, 99) (6.51, 95) (8, 95)]. The column temperature was maintained at 50 °C using a column heater. Under these experimental conditions, the LLOQ was 0.4 ng/mL.

All animals were maintained under standard housing conditions with access to standard pelleted food and water ad libitum throughout experiments. Animal experiments were conducted in accordance with Swiss national animal welfare regulations, under the ethically approved animal experimentation licenses authorized by the Cantonal Veterinary Authority of Basel city and the Federal Veterinary Office of Switzerland.

## Results

### Design and Synthesis

As explained in the introduction, closely related analogues to peptide (2) were designed to obtain structurally related molecules having high solubility and permeability. The decrease in *N*-methylation was realized by exchanging the *N*-methylated Tyr residue in (2) for alternative residues with a standard non-methylated amide bond. To compensate for this increase in polarity by returning to a standard amide bond, the phenolic OH of the Tyr was eliminated resulting in cyclic peptides bearing varying hydrophobic side chains (Fig. 1). As an overall effect, polarity was moved from the side chain to the backbone to enable a better shielding as compared to corresponding options for side chain functionalities. Thus, for the replacement of Tyr, we have foreseen residues Phe, Abu, and Ala resulting in peptides (3), (4), and (5) to assess the effects of bulk on the ability to shield the more polar amide bond, to monitor the influence on physico-chemical parameters like solubility, and finally to investigate pharmacokinetics (PK). The corresponding peptides were assembled on the solid phase and backbone cyclized in solution similar to procedures as reported earlier (Lewis et al. 2015).

### *In-silico* and In Vitro Profiling

**Fig. 2:**
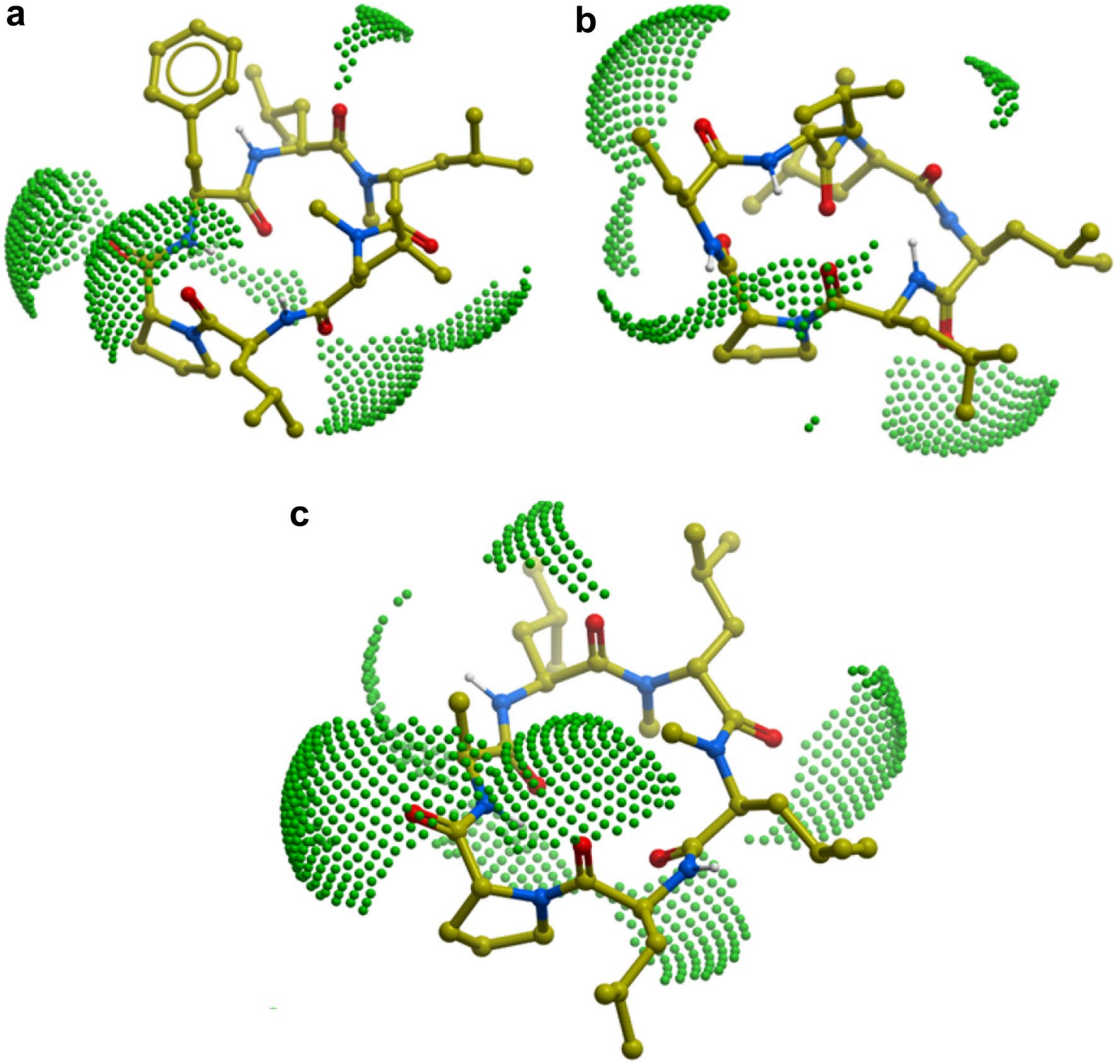
Representation of the solvent-accessible polar surface (*green dots*) for cyclic hexapeptides, **a** SAPSA of the most populated conformational cluster for peptide (3) [Phe-Leu-NMe-D-Leu-NMe-Leu-Leu-D-Pro], **b** SAPSA of most populated conformation cluster for peptide (4) [Abu-Leu-NMe-D-Leu-NMe-Leu-Leu-D-Pro], **c** SAPSA of most populated conformation cluster for peptide (5) [Ala-Leu-NMe-D-Leu-NMe-Leu-Leu-D-Pro]

**Fig. 3:**
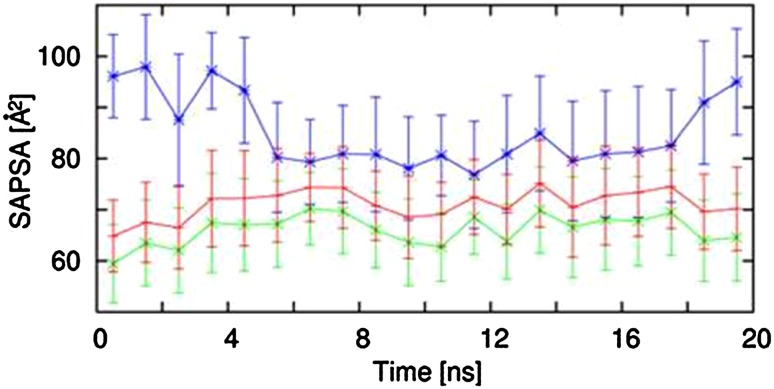
SAPSA during molecular dynamics simulation for peptide (4) in *green*, peptide (5) in *blue*, and the “truncated” peptide (4) corresponding to peptide (5) but using the trajectory of (4) in *red*; points and *error bars* show the mean and standard deviation for 1 ns averages, respectively

*In-silico* analyses were initiated in the first instance to determine the SAPSA, in particular for each of the 3 designed analogues. The SAPSA calculation differs from the classical PSA (Ertl et al. 2000), which is widely used to estimate the bio-availability of small molecules, in two aspects: (a) the area is computed for a solvent probe sphere in contact with nitrogen, oxygen, and their bound hydrogen atoms, rather than the surface on the atoms themselves; and (b) the surface is computed by averaging over an ensemble of 3-dimensional structures, instead of a sum of tabulated values of O and N atoms depending on their bond topology and independent of the 3-dimensional structure, in particular the actual solvent exposure. As a consequence, the SAPSA considers the average solvent-accessibility of polar atoms. Thus, this algorithm takes into account the ability of polar atoms to form intra-molecular hydrogen bonds or the effect of shielding from exposure to solvent by other apolar moieties. Comparing the SAPSA values for cyclic hexapeptides (3), (4), and (5), calculations indicate the Abu-peptide (4) with a SAPSA of 66 Å^2^ to be the most favorable compound, followed by the Phe-analogue (3) with a SAPSA of 70 Å^2^ (Table 1). In our experience, cyclic hexapeptides with a SAPSA of <80 Å^2^ are considered highly permeable, whereas above 150 Å^2^ only a low permeability is expected for this scaffold. The *in-silico* assessment illustrates that both aliphatic and aromatic shielding can be effective in masking polarity. The finding that the largest side chain, Phe in compound (3), which would be expected to lead to the best solvent shielding of the backbone, has not resulted in the lowest SAPSA highlights the fact that structural changes can have both an indirect and a direct effect on solvation. In the first case, slightly altered scaffold geometries result in a different conformer distribution and lead to changes with respect to the overall distribution of the polar surface area. In addition or alternatively, shielding by steric hindrance of solvent contacts has an influence on the SAPSA. In this study, introduction of a Phe residue is increasing the solvent shielding over an Ala in the same position, presumably by steric hindrance of solvent access. However, the predicted higher solvent accessibility of compound (3) in comparison with the Abu analogue (4) is expected to be derived from the influence on the conformer distribution of the peptide, shifting the equilibrium towards higher exposure of the backbone. According to structural clustering, the dominant conformations of peptides (3)–(5) as illustrated in Fig. 2a–c were obtained. The green colored solvent accessible polar surface area visually represents the exposed polarity. According to Table 1, the SAPSA could be substantially reduced compared to the starting structure (2), in particular for peptide (4). As a next step, we investigated further the Ala versus Abu side chain, due to the significant difference between these closely related peptides (4) and (5). In order to map the contribution of the side chain, the trajectory of peptide (4) (20 ns, 20,000 conformations stored) was modified to replace the terminal CH_3_ of the Abu side chain by a hydrogen atom. Effectively, on the basis of the scaffold geometry of peptide (4), a modified trajectory for peptide (5) was obtained followed by SAPSA calculation. Figure 3 shows in red the SAPSA values, averaged in intervals of 1 ns, for this modified trajectory mimicking peptide (5). The corresponding SAPSA values are only slightly larger than those of peptide (4) in green. Throughout the simulation, values are considerably lower than for the original molecular dynamics of peptide (5) represented in blue. According to these results, the effect of the additional methyl-group in peptide (4) (Abu side chain) is almost entirely related to an influence on the scaffold’s conformation rather than caused by shielding polar atoms in the vicinity of the side chain.

**Table 1:** In vitro and physicochemical data on cyclic hexapeptides

Peptide	Sequence	SAPSA (Å^2^)	SFC (min)	Sol. (mM, pH = 6.8)	Log PAMPA (m/s)	Class	MDCK (10^− 6^ cm/ s)	MLM (µL/ min/mg)	PPB (%)
(1)	[Tyr-Leu-D-Leu-Leu-Leu-D-Pro-]	146 ± 14	4.8	0.151	<−6.4	L	2.0	95	92
(2)	[NMe-Tyr-Leu-NMe-D-Leu-NMe-Leu-Leu-D-Pro]	127 ± 10	4.1	0.026	<−4.6	H	11.4	63	>99
(3)	[Phe-Leu-NMe-D-Leu-NMe-Leu-Leu-D-Pro]	70 ± 10	2.9	0.030	−4.0	H	11.6	488	>99
(4)	[Abu-Leu-NMe-D-Leu-NMe-Leu-Leu-D-Pro]	66 ± 9	2.7	0.602	−4.3	H	4.0	324	>99
(5)	[Ala-Leu-NMe-D-Leu-NMe-Leu-Leu-D-Pro]	85 ± 13	2.8	>1.000	−4.6	H	2.7	654	>99

Next, we applied SFC-analysis to compare calculations related to polar surface area with experimentally derived data. The retention time on a SFC-column containing H-bond acceptors and donors has been demonstrated to reflect the exposure of polar groups in the apolar environment applied during the chromatography process (supercritical CO_2_) (Goetz et al. 2014a, b). Thus, calibrated SFC retention times can be compared to the SAPSA values obtained from molecular dynamics. As indicated in Table 1, the ranking according to the *in-silico* assessment agrees very well with the measured SFC retention time. As a result, shielding of polar atoms under SFC conditions is realized most optimally in the Abu derivative (4) leading to a short retention time of 2.7 min. Interestingly, the trade-off - elimination of the phenol to reduce polarity and to reduce the degree of *N*-methylation–led to excellent solubility values for the new derivatives (4) and (5) incorporating aliphatic side chains (Table 1). In addition, the results from the PAMPA assay agree quite well indicating high permeability for peptides (2)–(5). However, in this case the best permeability was attributed to the Phe analogue. MDCK data are less consistent, but also suggest high permeability for peptide (3) and are broadly in agreement with the other assessments. However, most likely in the case of peptide (5), for which the MDCK assay is not aligned with PAMPA and SFC, the low predictability for oral uptake would have excluded this candidate from an in vivo experiment. Taking into account the in vitro profiling, it seems possible to identify cyclic peptides with good permeability and solubility.

In order to make predictions of pharmacokinetic properties of these cyclic peptides, further in vitro evaluation was carried out to determine plasma-protein binding (PPB) and metabolic stability in mouse liver microsomes (MLM). In terms of PPB, only the low-permeable, non-methylated analogue (1) showed a reduced plasma-protein binding of 92%, which can be attributed to its more polar characteristics (Table 1). The highly permeable tri-*N*-methylated analogue (2) and the three di-*N*-methylated analogues (3), (4), and (5), all showed very high plasma-protein binding >99%, and thus these peptides could not be discriminated between each other. In order to predict clearance in vivo, evaluation in vitro was assessed in MLM. The *N*-methylated compound (2) showed medium clearance (CL), while the non-methylated peptide, although poorly permeable, had a higher microsomal CL (Table 1). The di-*N*-methylated compounds (3), (4) and (5) all showed very high CL in the MLM assay, and for the Ala-derivative (5) the highest in vitro clearance was observed.

### I.v. and Per os (p.o.) Study in Mice: Pharmacokinetics of the Cyclohexapeptides

**Fig. 4:**
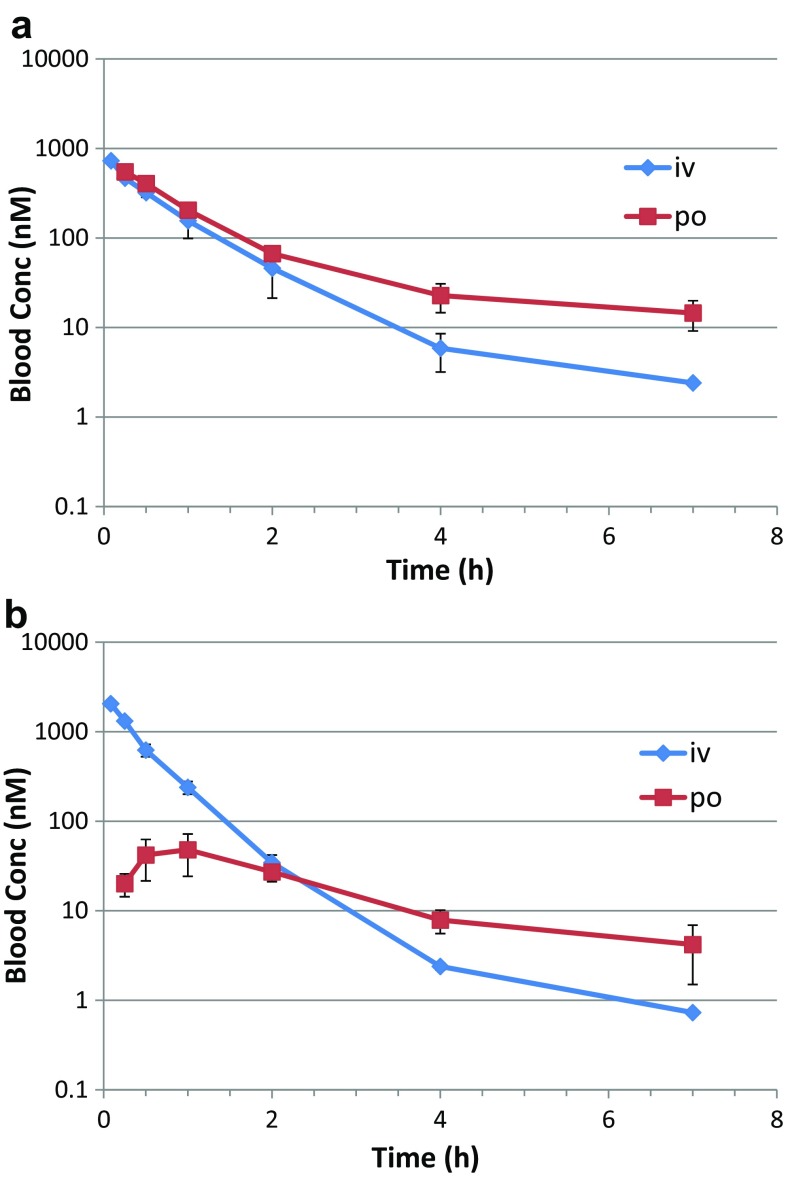
**a** Blood concentration i.v. and p.o. from mouse PK study on peptide (4). Data are mean ± standard deviation (n = 3 animals), **b** Blood concentration i.v. and p.o. from mouse PK study on peptide (5) Data are mean ± standard deviation (n = 3 animals)

The results of the in vivo p.o. study in mice confirmed the permeability predicted from the in vitro data for peptides (4) and (5) and for the others only to some degree (Table 2). As a result from the in vivo experiments in mice, in Table 2 three classes for in vivo CL can be identified. The low CL group consists of compounds (2) and (3), and one medium CL candidate, compound (4) with 49 mL/min/Kg was identified. The high CL group consists of compounds (1) and (5) with 82 and 105 mL/min/Kg, respectively (Table 2). In this context it is noteworthy to indicate, that mouse hepatic blood flow is around 90 mL/min/kg. In the medium CL group, compound (4) achieves the best oral %BAV (bioavailability on p.o. administration) of 39% suggesting permeability can compensate to some extent for clearance. The i.v. and p.o. profiles of this Abu-peptide (4) and the Ala-derivative (5) are provided in Fig. 4a, b. Surprisingly, the Phe-peptide (3) shows only a 7% oral %BAV, although it is arguably more permeable in terms of PAMPA and MDCK. In addition, this peptide exhibits a low CL of only 2 mL/min/kg. Potentially, its %BAV may have been impacted by the low solubility of only 0.03 mM. In this case, in vitro profiling, in particular the MDCK assay, tends to over-predict the % oral BAV (Table 1). In the high clearance group, only compound (5) with a CL of 105 mL/min/Kg achieves a medium absolute oral bioavailability of 23%, and has an excellent solubility of >1 mM. Interestingly, although the AUC p.o. is lower for the Abu-peptide than the Phe-analogue, the Cmax for peptide (4) is significantly higher compared to the Ala- and Phe-analogue (Table 2). In general we would conclude, that the MLM assessment does not necessarily reflect the in vivo outcome, although the ranking for cyclic peptides (3), (4) and (5) regarding CL are in line with in vitro and in vivo data. Overall, the mouse PK studies add more data on very much related peptides as previously studied in mice (Lewis et al. 2015) and rats (White et al. 2011), and demonstrate, that good oral bioavailability and solubility within one molecule can be achieved.

**Table 2:** PK parameters as determined in male OF1 mice for peptides (1)–(3), or C57BL/6 mice for compounds (4)–(5) (i.v. and p.o. each 3 animals)

Peptide	Sequence	CL Mouse (mL/min/Kg)	% oral BAV	Vss (L/kg)	T1/2term (h)	MRT (h)	AUC i.v. d.n. (nM h)	AUC p.o. d.n. (nM h)	Cmax d.n. (nM)	Tmax (h)
(1)	[Tyr-Leu-D-Leu-Leu-Leu-D-Pro-]	82 ± 15	2 ± 1	0.8 ± 0.2	0.2 ± 0.0	0.2 ± 0.0	292 ± 62	6 ± 2	3 ± 2	0.6 ± 0.3
(2)	[NMe-Tyr-Leu-NMe-D-Leu-NMe-Leu-Leu-D-Pro]	1 ± 0	30 ± 2	0.4 ± 0.0	22.6 ± 5.4	9.6 ± 0.4	29,618 ± 2975	8967 ± 706	534 ± 72	3.8 ± 3.1
(3)	[Phe-Leu-NMe-D-Leu-NMe-Leu-Leu-D-Pro]	2 ± 0	7 ± 2	0.6 ± 0.1	5.5 ± 0.4	6.1 ± 0.1	13,508 ± 1557	896 ± 325	77 ± 9	0.3 ± 0.1
(4)	[Abu-Leu-NMe-D-Leu-NMe-Leu-Leu-D-Pro]	49 ± 14	39 ± 4	2.1 ± 0.2	0.8 ± 0.2	0.8 ± 0.3	544 ± 176	214 ± 24	183 ± 10	0.3 ± 0.0
(5)	[Ala-Leu-NMe-D-Leu-NMe-Leu-Leu-D-Pro]	105 ± 26	23 ± 4	2.8 ± 0.3	0.4 ± 0.1	0.5 ± 0.2	257 ± 70	59 ± 10	62 ± 9	0.3 ± 0.0

### Hepatic Portal Vein (H.P.V.) Study in Rat: Pharmacokinetics of the Cyclohexapeptides

**Fig. 5:**
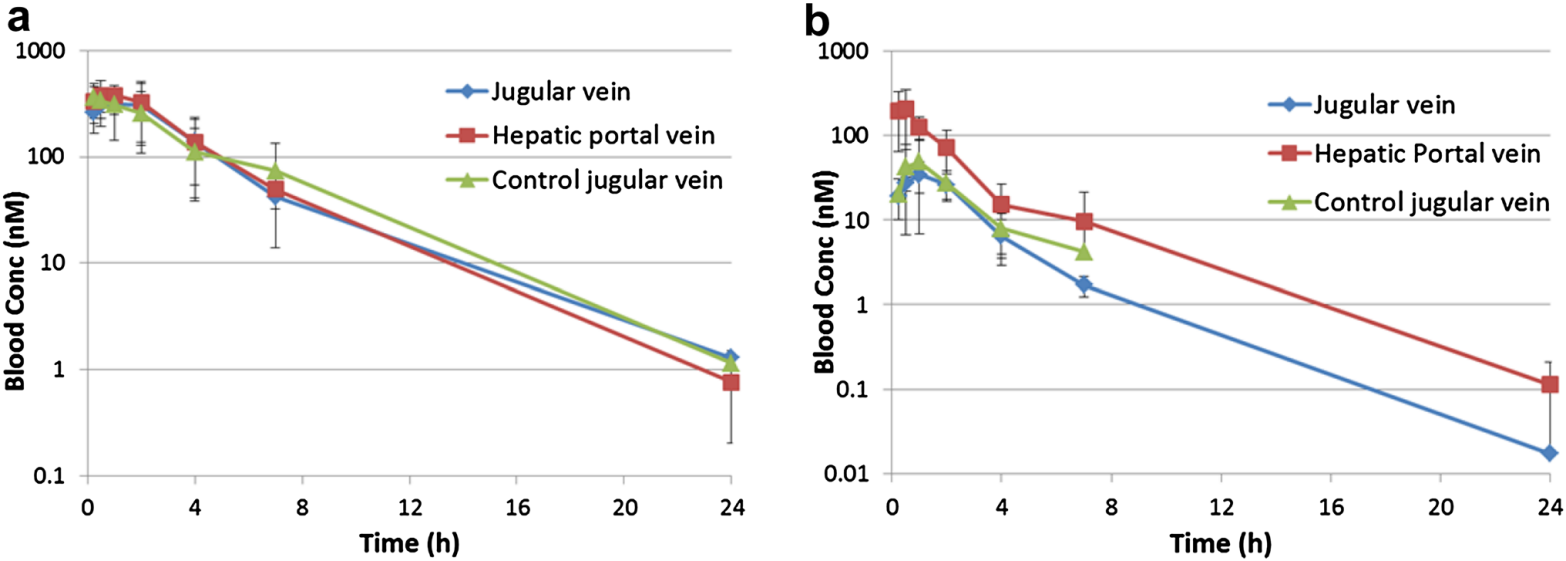
**a** Compound (4) [Abu-Leu-NMe-D-Leu-NMe-Leu- Leu-D-Pro] H.P.V study and control jugular vein study, **b** Compound (5) [Ala-Leu-NMe-D-Leu-NMe-Leu-Leu-D-Pro] H.P.V study and control jugular vein study (the 24 h point for control jugular vein was below the LLOQ)

In a follow-up study, we intended to expand our understanding regarding in vivo clearance. In this context, we considered assessing the role of liver metabolism, and in particular the first pass effect, in a model similar to that reported recently (Holenarsipura et al. 2015). Accordingly, we decided to conduct a hepatic portal vein (H.P.V.) study in Sprague Dawley rats due to the technical difficulties associated with a smaller species like mice. Rats were equipped with double jugular vein and hepatic portal vein cannulation. A standard jugular vein cannulation experiment was run in parallel as a control. The study was designed to measure peptide concentrations in the hepatic portal vein after absorption by the intestine before any liver metabolism could have commenced and to compare these values to the peptide concentration measured in circulation. Since we were intrigued with the difference in CL between the two closely related compounds (4) and (5), these peptides were considered for the H.P.V. study in the first instance, as both exhibit good solubility properties. In order to avoid ambiguity, which may result from differential stability as a result of proteolytic events, for compounds (1)–(5) stability studies were carried out in blood, kidney, and liver cell homogenate (see Supplementary Figures S3 A-E). All five cyclic peptides proved to be stable under these conditions, and as indicated above, peptides (4) and (5) were selected for the H.P.V. experiment. As illustrated in Fig. 5a, b, there is excellent reproducibility for the control jugular vein study compared to the H.P.V. study in terms of exposure. With respect to the reduction of exposure resulting from the liver first pass effect, it is easy to discriminate between the two cyclohexapeptides (4) and (5) (Table 3). For the Abu derivative (4), only a moderate metabolism is observed (11% first pass effect), while the Ala-peptide shows a 77% liver first pass effect. The high exposure in the portal vein for the Abu-analogue (4), reflected in the AuC (area under the curve) value of 1839 nM/h, can be attributed to its increased permeability as discussed above. The clearance and %BAV determined in this experiment fit quite well with the previous mouse study. This clear-cut result with respect to the pronounced difference in the first pass liver effect came as a surprise, since both cyclic peptides studied differ only by one methylene group. In addition, a pronounced effect regarding liver metabolism has not been reported for this type of molecules. The differential behavior of peptides (4) and (5) may be explained by subtle effects of the side chain on the altered scaffold geometry, and obviously has a profound effect on the in vivo pharmacokinetics of these two compounds.

**Table 3:** Summary of data from a single rat hepatic portal vein experiment (dose i.v. and p.o.: 3 mg/kg)

Peptide	Sequence	Portal vein (nM/h)	Jugular vein (nM/h)	CL Rat (mL/min/Kg)	Liver first pass effect (%)
(4)	[Abu-Leu-NMe-D-Leu-NMe-Leu-Leu-D-Pro]	1839	1656	41	11
(5)	[Ala-Leu-NMe-D-Leu-NMe-Leu-Leu-D-Pro]	449	104	70	77

## Discussion

Cyclic peptides offer a large spectrum of opportunities for the discovery of novel therapeutics (Whitty et al. 2016; Lewis et al. 2015; White et al. 2011), particularly considering difficult targets like protein–protein interactions, for which small molecule inhibitors with sufficient affinity and specificity are difficult to identify. By extending the chemical space of ligands beyond the 500 MW citerion, cyclic peptides are able to target binding surfaces that are more flat and less productive in terms of binding efficiency than, for example, the enzymatic pocket of a kinase or protease. However, macromolecules also encounter important challenges in the optimization of their “drug-like” properties, e.g. permeability, and in particular oral bioavailability. In the chemical space of molecules beyond the “rule-of-five” (Veber et al. 2002; Lipinski et al. 2001), it has been shown that the shielding or “masking” of polarity (Biron et al. 2008) can lead to significant improvement in permeability and as a consequence also lead to an enhancement of oral uptake.

As mentioned in the introduction, various strategies to reduce polar surface area have been investigated. *N*-methylation has been applied for quite some time (White et al. 2011; Nielsen et al. 2012; Beck et al. 2012; Rand et al. 2012; Alex et al. 2011), but only recent contributions have demonstrated for cyclic penta- and hexaleucine peptides, that oral absorption can be achieved even in the absence of* N*-Methylation (Hill et al. 2014). In this special case, shielding was achieved by incorporation of bulky amino acids. In addition, NMR (Wang et al. 2014) has been shown to be a valuable tool to verify intramolecular hydrogen bonds, which are of prime importance in shielding polarity and in evaluating the effect of* N*-methylation on conformation. Design, and production of libraries inspired by peptidic natural products such as Sanguinamide A (Nielsen et al. 2012), Guangomide A and Phepropeptide (Nielsen et al. 2014; Hewitt et al. 2015; Schwochert et al. 2016) have also been demonstrated to be a promising strategy for the identification of permeable peptides and understanding the underlying principles. In particular, recent investigations on cyclic peptides derived from Sanguinamide A resulted in a molecule exhibiting solvent-dependent flexibility and improved permeability (Bockus et al. 2015). In addition, albeit only on the basis of Caco-2 data, peptoid-type modifications, described from the same group, may offer more opportunities to add on polar side chains (Furukawa et al. 2016). Furthermore, new methods such as SFC (Goetz et al. 2014a, b) and the computational “solvent accessible polar surface area” (Lewis et al. 2015) provide more opportunities to support the design of permeable macrocycles. In addition to permeability, however, proteolytic degradation and metabolism are key aspects in determining oral bioavailability. In contrast to their linear counterparts, macrocyclic peptides (Kwon and Kodadek 2007) have more restricted conformational freedom. Firstly, this contributes to an increased proteolytic stability. Secondly, cyclization, due to the absence of exposed termini reduces the polarity, and therefore, the likelihood for intra-molecular hydrogen bonding (White et al. 2011) is increased.

Embarking from compounds (1) and (2), which have been extensively discussed in our previous publication (Lewis et al. 2015), we established the differential effects of hydrophobic shielding. As a result, for the in vitro profiling as well as for the in vivo experiments, subtle conformational changes play an important role for the properties of the side chain variations investigated. Interestingly, the SFC retention time and PAMPA values correlate quite well with the observed oral bioavailability in mice and rats, however, there is a need for additional assays to enable a better in vivo predictability. This statement also holds true for the assessment of metabolic stability by liver microsomal assays, which may expose an unnatural high proteolytic activity, and for this reason, may not really reflect the in vivo situation. Furthermore, the PK studies clearly pointed out differences in oral uptake, which was gratifying as we could not necessarily anticipate identifying analogues with one less *N*-methyl, good solubility, and an oral bioavailability >30%. The pronounced difference in clearance for the Ala versus the Abu analogue prompted us to conduct the H.P.V. experiment. While the differential permeability, reflected by the increased exposure for the Abu peptide, could be rationalized based on the in vitro profiling, the drastic first liver pass effect for the Ala peptide came as a surprise. We speculate that Cytochrome P450 enzymes hold responsible for this phenomenon, and we are looking forward to studying the oxidative metabolism of compound (5). In principle, lower AUC values following oral administration in the in vivo experiments, especially for peptide (5), could also result from intestinal Cytochrome P450 activity. As a next step, we foresee the identification of the metabolites for these cyclic peptides, in order to learn what features are important for processing and how metabolization can be modulated.

## Conclusions

Previous reports demonstrated how multiple *N*-methylation and matched hydrogen bond pairs across the macrocycle (White et al. 2011; Nielsen et al. 2014; Fouche et al. 2016a, b) could improve oral bioavailability. In this study, we have shown how hydrophobic shielding can contribute to masking polarity, and thereby enhancing permeability, while simultaneously providing excellent solubility. Thus, balancing permeability with other physico-chemical parameters has been successfully demonstrated in this study, while increasing passive permeability often occurs at the expense of solubility and lipophilicity as indicated in a recent publication (Thansandote et al. 2015). However, changes in the overall scaffold geometry as a result of minute alterations in a particular side-chain have to be considered, especially if the design of improved features targets only local improvements. In addition to in vitro validation (Lewis et al. 2015; White et al. 2011), the results presented in this contribution clearly demonstrate the significant role of liver metabolism, and how small structural changes fundamentally influence in vivo effects. In the case of the Abu substitution, both enhanced permeability and the more limited metabolism in the liver lead to good oral exposure and bioavailability. Another aspect relates to the role of transporters regarding oral uptake, and in addition, the potential for chiral recognition. A systematic study provided evidence for selective carrier-mediated transport based on Caco-2 permeability measurements (Marelli et al. 2015). However, shortcomings of this type of cellular assays have been recognized, and it seems there is some potential for intestinal organoids to enable establishing an assay for assessing uptake more in line with physiological conditions (Zietek et al. 2015). Any assays or combinations thereof enabling the prediction of intestinal permeability and metabolic stability would be of assistance in encouraging advances in terms of oral exposure. Having gained a more comprehensive understanding of both permeability and metabolism for cyclic peptides, further experiments on other scaffolds are needed to assess the contribution of liver metabolism to clearance, in particular for peptides designed for oral bioavailability. Interestingly, in this case the more permeable peptide (4) is also more resistant to liver metabolism and the question may arise, whether this situation is an exception or more a standard case. Furthermore, it will be important to know, whether there are larger permeable peptides, which due to their size may not be metabolized anymore. Acquiring a more in-depth understanding of the various processes, in particular correlating structural features with the amount of metabolization, would definitely help in designing modifications with increased metabolic stability. In summary for peptides able to penetrate membrane barriers, we propose investigating liver metabolism at an early point in time during therapeutic peptide development.

## Electronic supplementary material

Below is the link to the electronic supplementary material.

Supplementary material 1 (DOCX 1815 KB)

## Abbreviations

ADME: Absorption, distribution, metabolism, and excretion
AUC: Area under the curve
%BAV: Bioavailability on p.o. administration
CL: In vitro or in vivo clearance
Cmax: Observed maximum concentration in blood
d.n.: Dose normalized
H.P.V: Hepatic portal vein
i.v.: Intravenous
LLOQ: Lower limit of quantification
MDCK: Madin Darby canine kidney cells
MLM: Mouse liver microsomes
MRT: Mean residence time
PAMPA: Parallel Artificial Membrane Permeability Assay
PK: Pharmacokinetics
PPB: Plasma-protein binding
rmsd: Root mean squared deviation
p.o.: Per os
PSA: Polar surface area
SAPSA: Solvent-accessible polar surface area
SFC: Supercritical fluid chromatography
T_1/2_ term: Terminal half-life for elimination
Tmax: Time of observed maximal blood concentration
Vss: Apparent volume of distribution at steady state
