# Supplementary material for: Modulation of Oral Bioavailability and Metabolism for Closely Related Cyclic Hexapeptides
Source: Int J Pept Res Ther. 2017 Mar 28;24(1):35–48. doi: 10.1007/s10989-017-9590-8 (PMC5838147; doi:10.1007/s10989-017-9590-8)
Supplement: Supplementary file 1 — Supplementary material 1 (DOCX 1815 KB) [file 10989_2017_9590_MOESM1_ESM.docx]

**Modulation of oral bioavailability and metabolism for closely related cyclic hexapeptides**

**Thomas Vorherr, Ian Lewis, Joerg Berghausen, Sandrine Desrayaud,**

**and Michael Schaefer**

**Novartis Institutes for Biomedical Research, CH-4002 Basel, Switzerland**

thomas.vorherr@novartis.com

**Supplementary Information**

**Analytical Methods**

UPLC-MS Standard Peptide

Waters  Acquity UPLC with a Xevo G2-S QTof MS; Column: Acquity UPLC CSH C18, 2.1 x 100mm Column, 1.7 µm; Column Temperature: 80°C; Eluents: A: water + 0.05% TFA; B: acetonitrile + 0.04% TFA; Gradient: initial 5% B; from 5 % to 98 % B in 9.4 min; 1.0 min 98 % B; Flow rate: 0.5 mL/min; Injection Mode: Partial loop; Compound (1), compound (2), compound (3), compound (4)

UPLC-MS Non-Polar Peptide

Waters  Acquity UPLC with a Xevo G2-S QTof MS; Column: Acquity UPLC BEH C4, 2.1 x 100mm Column, 1.7 µm; Column Temperature: 80°C; Eluents: A: water + 0.05% TFA; B: acetonitrile + 0.04% TFA; Gradient: initial 5% B; from 5 % to 98 % B in 9.0 min; 0.5 min 98 % B; Flow rate: 0.5 mL/min; Injection Mode: Partial loop; compound (5).

**UPLC-MS Results**

| Fig. S1 UPLC-MS Data Compound (1). – (5). | |
| --- | --- |
| Fig. S1A Compound (1).  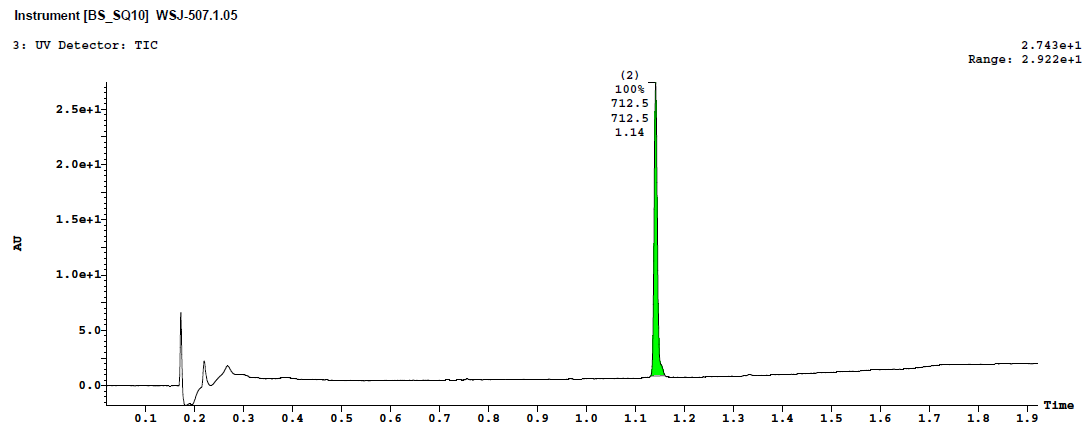 | Fig. S1B Compound (1): +ve and –ve mode  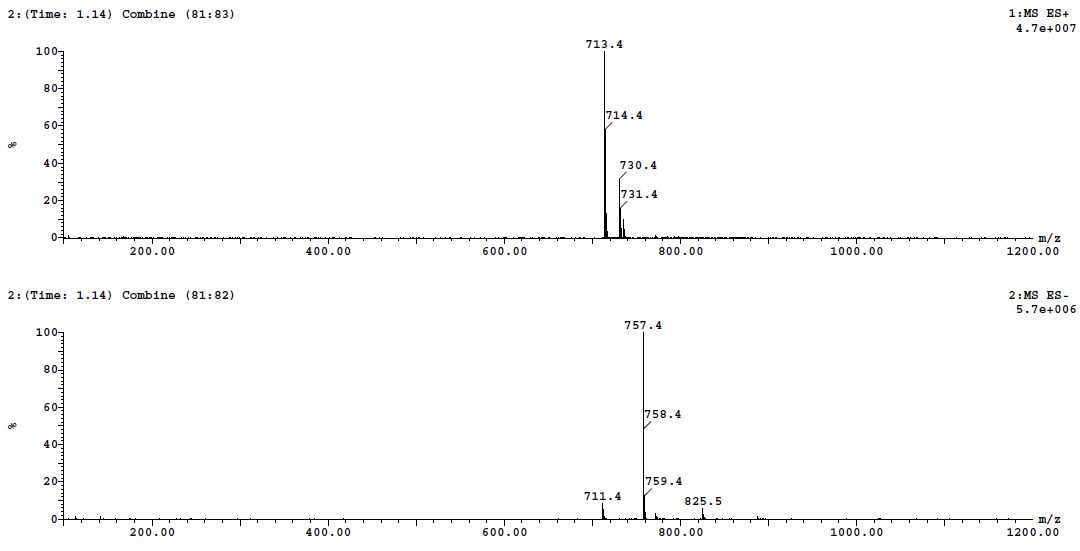 |
| Fig. S1C Compound (2).  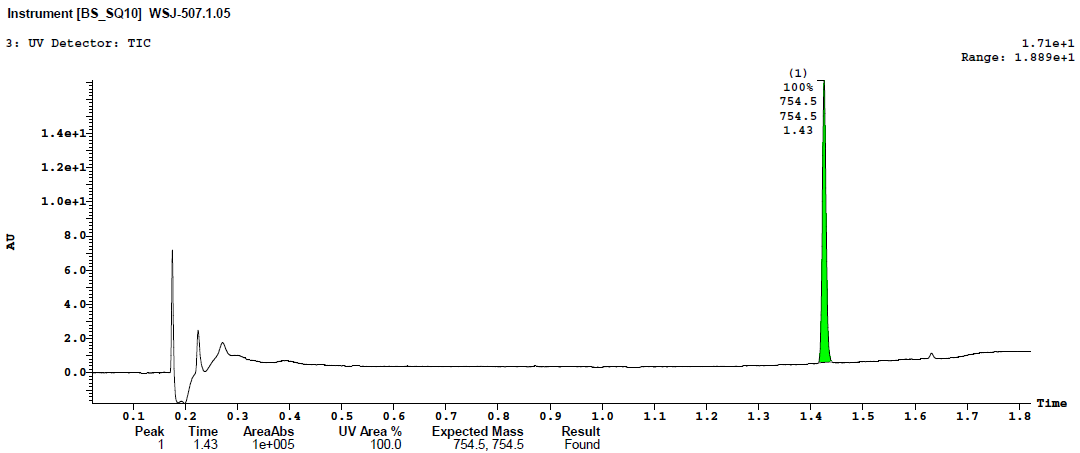 | Fig. S1D Compound (2): +ve and –ve mode  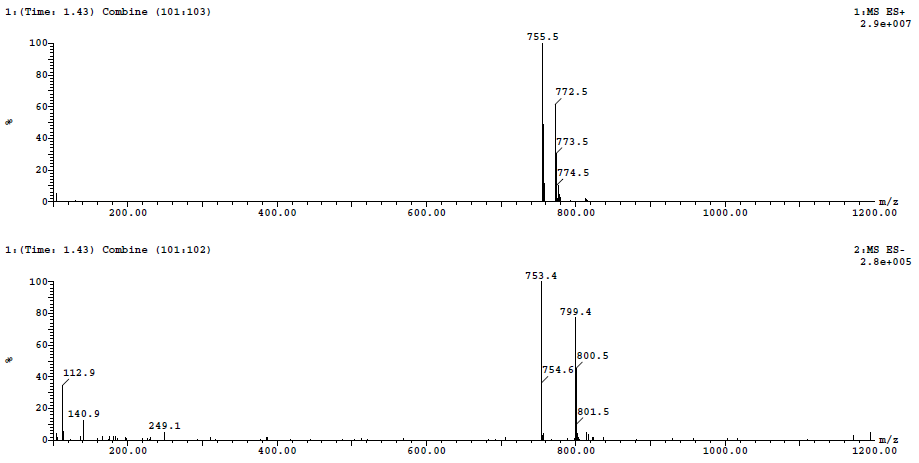 |
| Fig. S1E Compound (3).  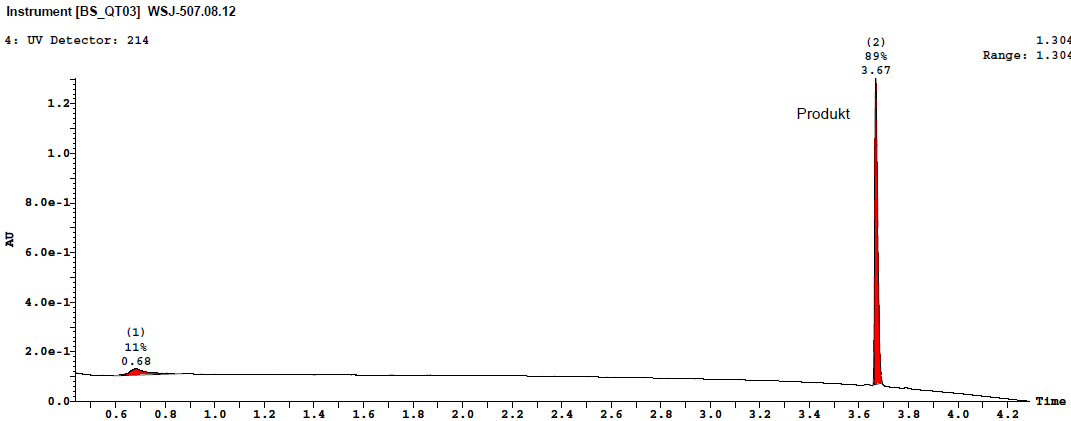 | Fig. S1F Compound (3): +ve and –ve mode  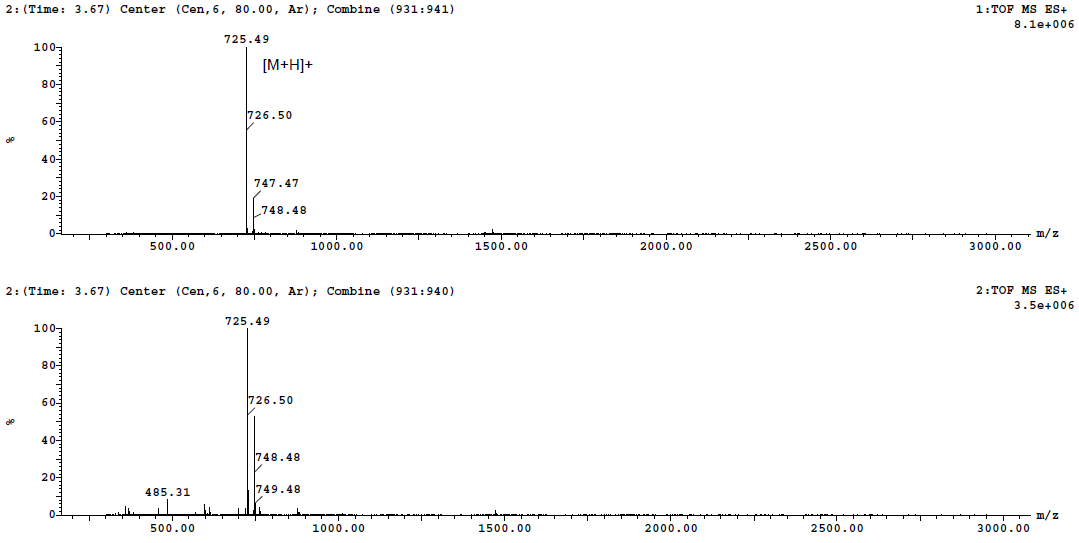 |
| Fig. S1G Compound (4).  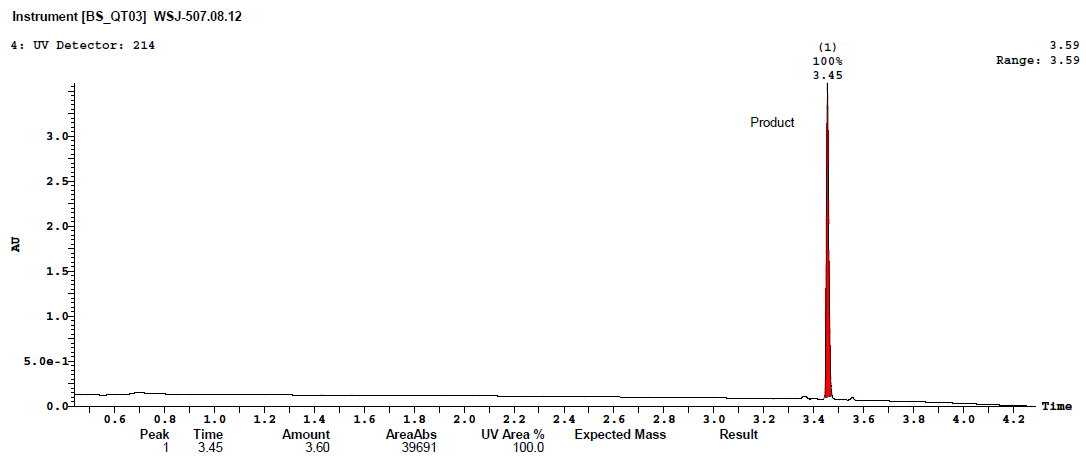 | Fig. S1H Compound (4): +ve and –ve mode  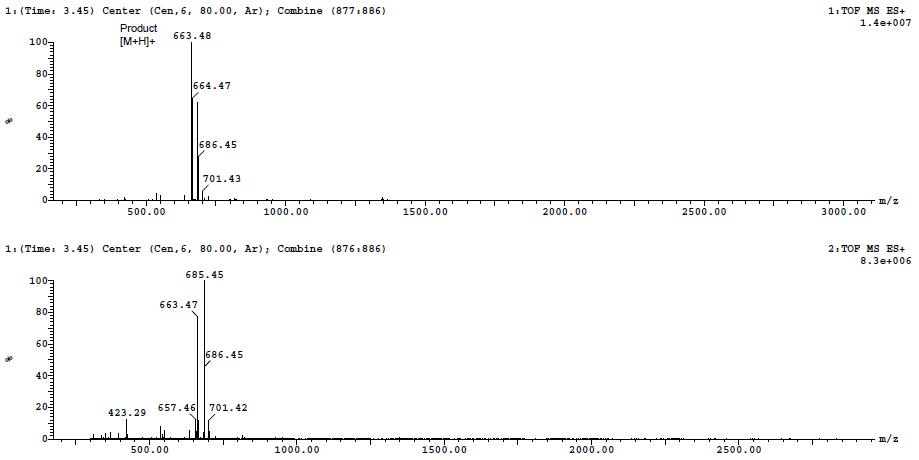 |
| Fig. S1I Compound (5).  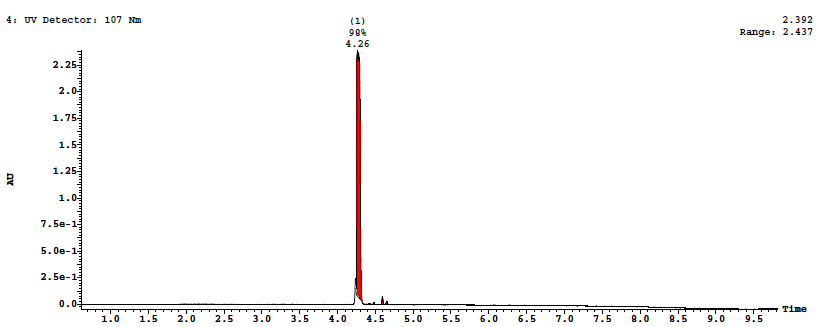 | Fig. S1J Compound (5): +ve and –ve mode  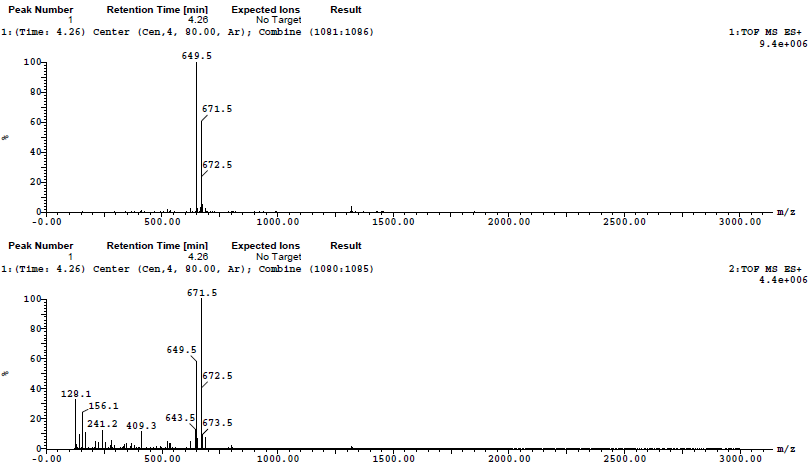 |
| Table S1 Summary table: UPLC-MS Data: compounds (1) – (5). | |
| \| **Sample** \| **Formula** \| **Ion observed** \| **Calculated** \| **Measured** \| \| --- \| --- \| --- \| --- \| --- \| \|  \|  \|  \|  \|  \| \|  \|  \|  \|  \|  \| \| Compound 1 \| C_38_H_60_N_6_O_7_ \| [M+H]^+^ \| 713.4 \| 713.4 \| \|  \|  \|  \|  \|  \| \| Compound 2 \| C_41_H_66_N_6_O_7_ \| [M+H]^+^ \| 755.5 \| 755.5 \| \|  \|  \|  \|  \|  \| \| Compound 3 \| C_40_H_64_N_6_O_6_ \| [M+H]^+^ \| 725.5 \| 725.5 \| \|  \|  \|  \|  \|  \| \| Compound 4 \| C_35_H_62_N_6_O_6_ \| [M+H]^+^ \| 663.5 \| 663.5 \| \|  \|  \|  \|  \|  \| \| Compound 5 \| C_34_H_60_N_6_O_6_ \| [M+H]^+^ \| 649.5 \| 649.5 \| | |

**NMR Spectroscopy**

**Sample Preparation**

1 mg of each lyophilized peptide was dissolved in either 40 μl d6-DMSO or 40 μl de-acidified CDCl_3_, vortexed for 20 s and transferred into 1.7 mm SampleJet NMR sample tubes. Tubes were then inserted into 1.7 mm shuttles and placed on the automatic sample changer.

**Data Acquisition** **& Processing**

^1^H detected 1D and 2D NMR spectra were obtained using a Bruker 600 MHz AVANCE III HD spectrometer equipped with a 1.7mm TCI cryo probe and a z-gradient system. 1D proton spectra were recorded with a standard one-pulse sequence (30 degree flip angle) with a relaxation delay of 2.5 sec and an acquisition time of 2.73 sec. 16 scans of 65536 points covering 12019.23 Hz were recorded. Data was zero-filled to 65536 complex points and an exponential window function was applied with a line-broadening factor of 0.3 Hz prior to Fourier transformation.

All 2D experiments for NMR assignment were recorded at a temperature of 300K with a relaxation delay of 1.5 s except the TOCSY experiment which has a relaxation delay of 1 s. For gradient COSY spectra (von Kienlin et al. 1991; Carpenter et al. 1992) a data matrix of 1024 x 2048 points covering 6602.1 x 6602.1 Hz was recorded with 2 scans for each increment. Data was linear predicted to 2048 x 2048 points using 32 coefficients and zero filled to 2048 x 4096 points. A sine square bell shaped window function was applied in F2 and a cosine square bell shaped window function in F1, respectively, prior to magnitude mode type 2D Fourier transformation. For edited coherence order selective gradient HSQC spectra (Kay et al. 1992; Parella et al. 1997) using adiabatic inversion pulses on the carbon channel (Kupce 2001), a data matrix of 256 x 2048 points covering 24901 x 8417.5 Hz was recorded using 2 scans for each increment. Data was linear predicted to 1024 x 2048 points using 60 coefficients and zero filled to 2048 x 2048 points prior to echo-anti echo type 2D Fourier transformation. A sine square bell shaped window function shifted by π/2 in both dimensions was applied. For HMBC spectra (Bax and Summers 1986) a data matrix of  512 x 4096 points covering 33805 x 9014.4 Hz with 4 scans for each increment was recorded using a double low pass J-filter and F1 absorption mode (Meissner and Sorensen 2000). Data was linear predicted to 1024 x 4096 points using 32 coefficients and zero-filled to 2048 x 4096 complex points prior to echo-anti echo type 2D Fourier transformation. A cosine square shaped window function was applied in F1 and a sine shaped window function shifted by π/4 was applied in F2. Data was converted to magnitude mode in F2 prior to analysis. ROESY spectra (Bax and Davis 1985a; Hwang and Shaka 1992; Thiele et al. 2009) with an effective field of γB1 = 8333 Hz and an spin lock time of 200 msec were recorded for a data matrix of 256 x 2048 points covering 8417.5 x 8417.5 Hz. 4 scans were recorded for each increment. Data was linear predicted to 512 x 2048 points using 32 coefficients prior to States-TPPI type 2D Fourier transformation and a sine square bell shaped window function shifted by π/2 in both dimensions was applied. For MLEV-17 based TOCSY spectra (Bax and Davis 1985b) a data matrix of 256 x 2048 points covering 8417.5 x 8417.5 Hz 2 scans were recorded for each increment. Data was linear predicted to 512 x 2048 points using 32 coefficients and zero filled to 1024 x 2048 points prior to States-TPPI type 2D Fourier transformation. A cosine square bell shaped window function was applied in both dimensions. All spectra were referenced according to the internal solvent signal (1H: d6-DMSO = 2.50 ppm and 13C: d6-DMSO = 39.52 ppm; 1H: CDCl_3_ = 7.26 ppm and 13C: CDCl_3_ = 77.16 ppm) (Gottlieb et al. 1997).

| **Abbreviation** | **Description** |
| --- | --- |
| COSY | Correlation spectroscopy |
| DAD | Diode array detector |
| ESI | Electrospray ionization |
| FWHM | Full width half maximum |
| HMBC | Heteronuclear multi-bond correlation |
| HPLC | High pressure liquid chromatography |
| HR | High resolution |
| HSQC | Heteronuclear single quantum coherence |
| IR | Infrared |
| LC | Liquid chromatography |
| LC/MS | Liquid chromatography/mass spectrometry |
| MS | Mass spectrometry |
| MTPA | Methoxytrifluoromethylphenylacetic acid |
| m/z | Mass to charge ratio |
| NMR | Nuclear magnetic resonance |
| NOE | Nuclear Overhauser effect |
| OS | Optical Spectroscopy |
| ROESY | Rotating frame Overhauser effect spectroscopy |
| ppm | Parts per million |
| TIC | Total ion chromatogram |
| 1D | One-dimensional |
| 2D | Two-dimensional |

**References**

Bax A, Davis DG (1985a) Practical Aspects of Two-Dimensional Transverse NOE Spectroscopy. J Magn Reson 63:207-213

Bax A, Davis DG (1985b) MLEV-17-based two-dimensional homonuclear magnetization transfer spectroscopy. J Magn Reson 65:355-360

Bax A, Summers MF (1986) 1H and 13C Assignments from Sensitivity-Enhanced Detection of Heteronuclear Multiple-Bond Connectivity by 2D Multiple Quantum NMR. J Am Chem Soc 108:2093-2094

Carpenter TA, Colebrook LD, Hall LD, Pierens GK (1992) Applications of Gradient-Selective COSY and DQCOSY to Brucine and Gibberellic Acid. Magn Reson Chem 30:768-773

Gottlieb HE, Kotlyar V, Nudelman A (1997) NMR Chemical shifts of Common Laboratory Solvents as Trace Impurities. J Org Chem 62:7512-7515

Hwang TL, Shaka AJ (1992) Cross Relaxation without TOCSY: Transverse Rotating-Frame Overhauser Effect Spectroscopy. J Am Chem Soc 114:3157-3159

Kay LE, Keifer P, Saarinen T (1992) Pure absorption gradient enhanced heteronuclear single quantum correlation spectroscopy with improved sensitivity. J Am Chem Soc 114:10663-10665

Kupce E (2001) Applications of adiabatic pulses in biomolecular nuclear magnetic resonance. Methods Enzymol 338:82-111

Meissner A, Sorensen OW (2000) Economizing spectrometer time and broadband excitation in small-molecule heteronuclear NMR correlation spectroscopy. Broadband HMBC. Magn Reson Chem 38:981–984

Parella T, Sánchez-Ferrando F, Virgili A (1997) Improved Sensitivity in Gradient-Based 1D and 2D Multiplicity-Edited HSQC Experiments. J Magn Reson 126:274-277

Thiele C.M, Petzold K, Schleucher (2009) EASY ROESY: Reliable Cross-Peak Integration in Adiabatic Symmetrized ROESY. Chem Eur J 15:585-588

von Kienlin M, Moonen CTW, van der Toorn A, van Zijl PCM (1991) Rapid Recording of Solvent-Suppressed 2D COSY Spectra with Inherent Quadrature Detection Using Pulsed Field Gradients. J Magn Reson 93:423-429

**NMR data**

| Fig. S2A Compound (1) CDCl3 | Fig. S2B Compound (1) d^6^-DMSO |
| --- | --- |
| 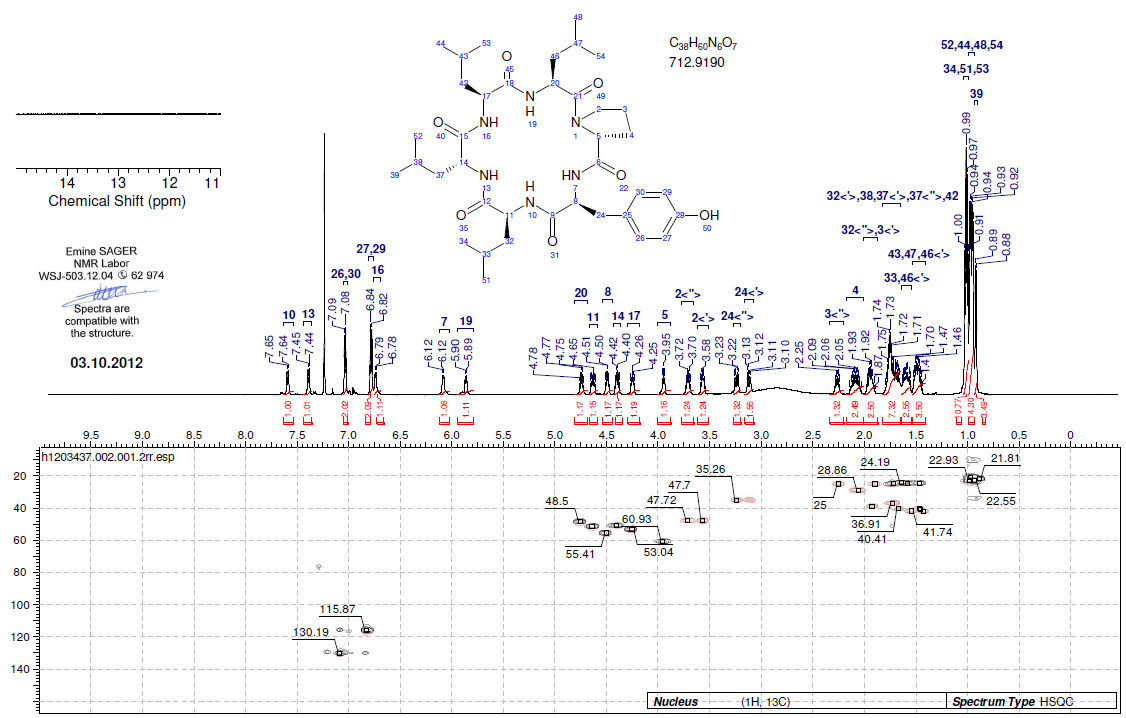 | 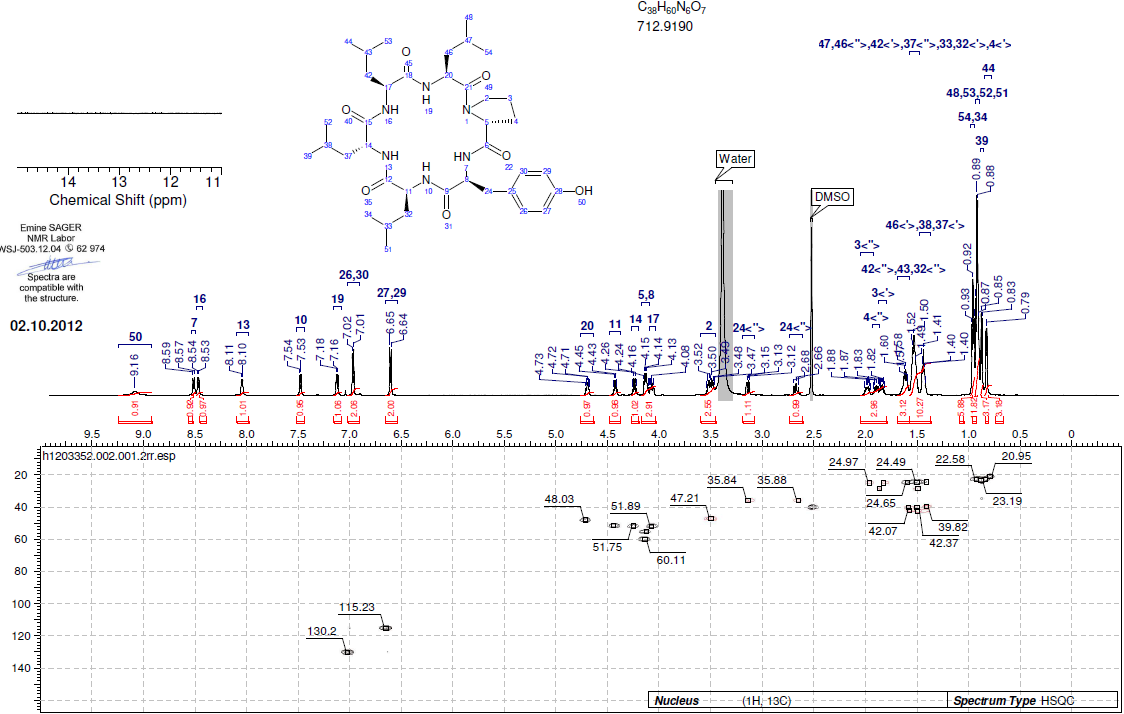 |
| Fig. S2C Compound (2) CDCl3 | Fig. S2D Compound (2) d^6^-DMSO |
| 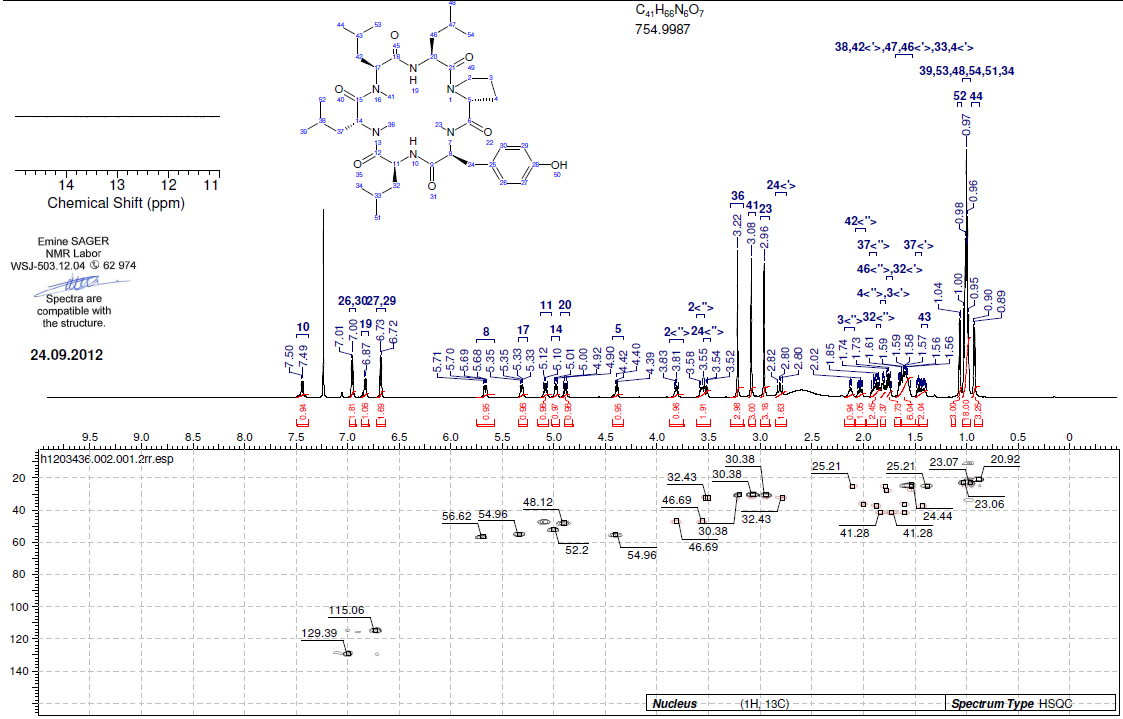 | 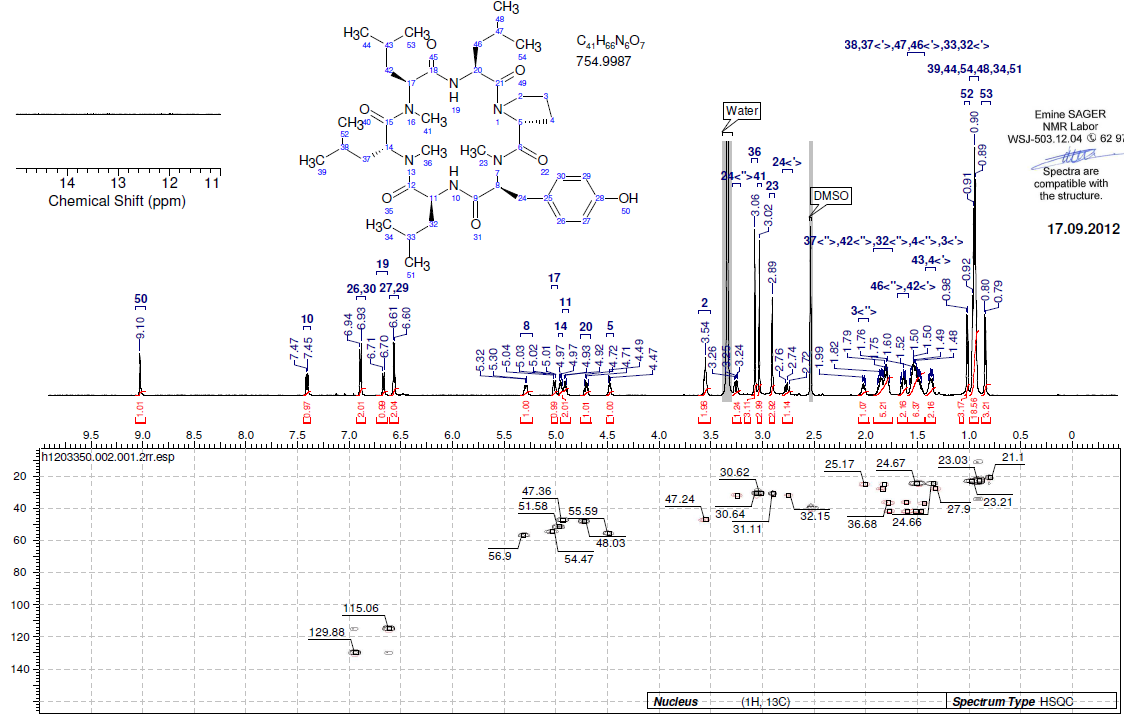 |
| Fig. S2E Compound (3) CDCl3 | Fig. S2F Compound (3) d^6^-DMSO |
| 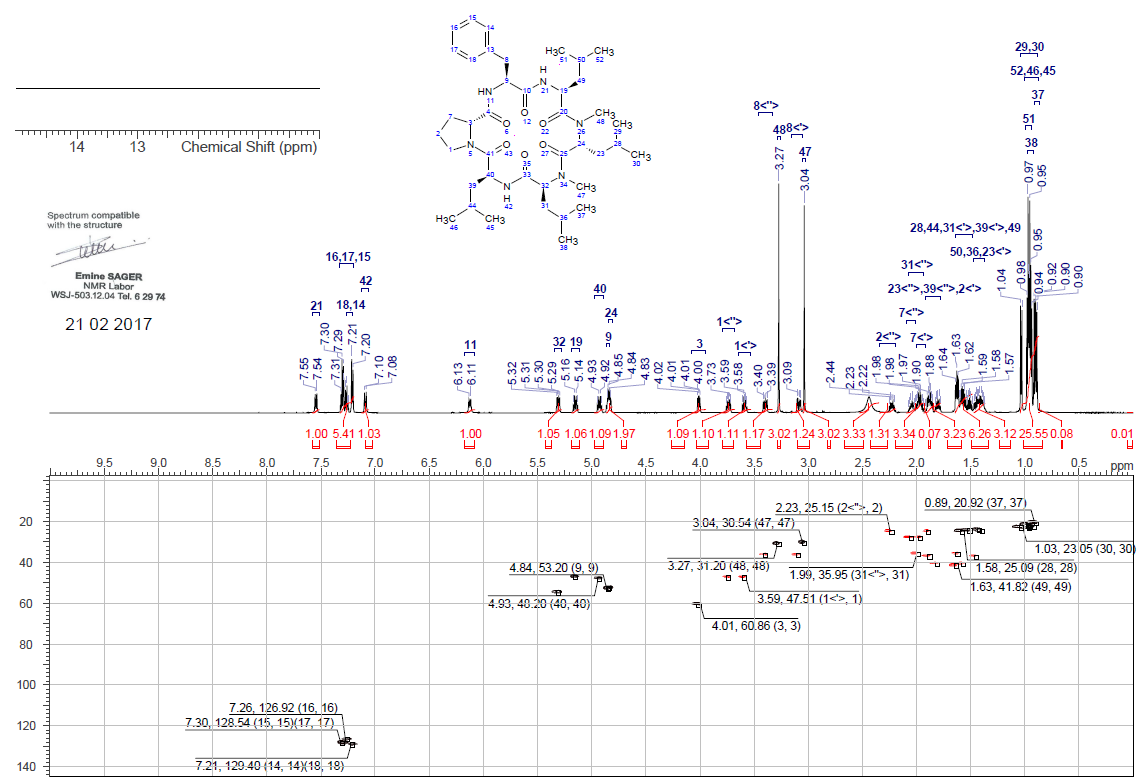 | 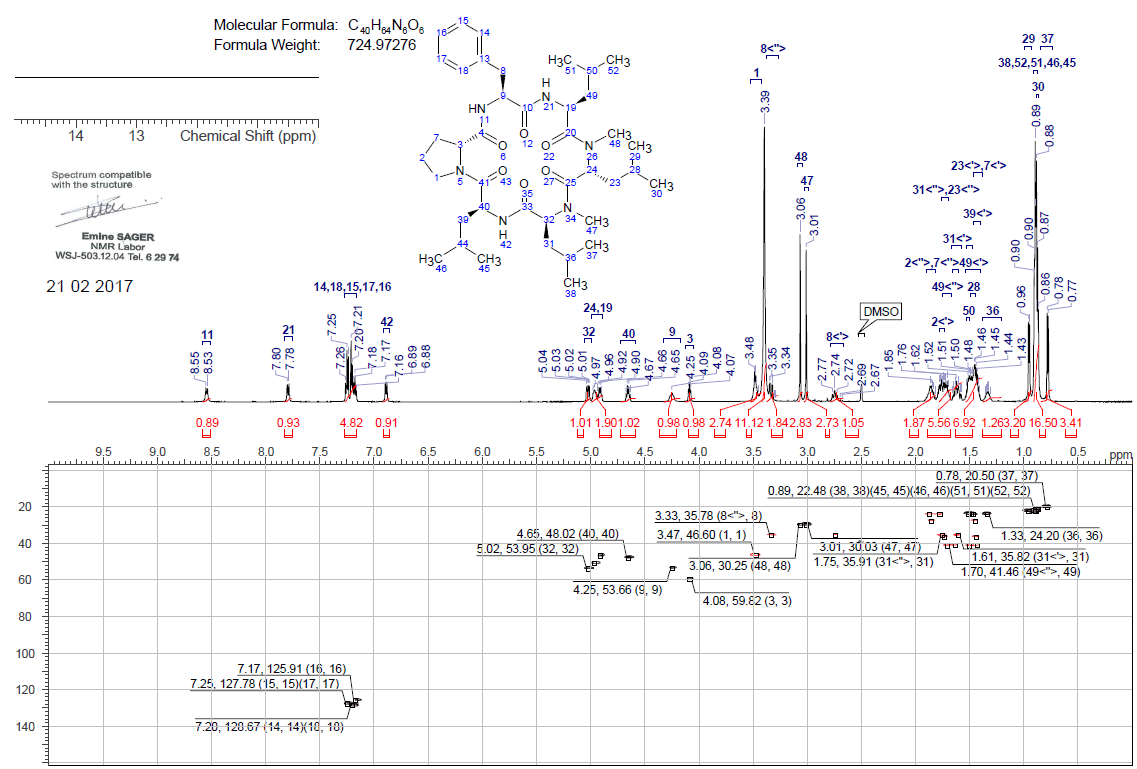 |

| Fig. S2G Compound (4) CDCl3 | Fig. S2H Compound (4) d^6^-DMSO |
| --- | --- |
| 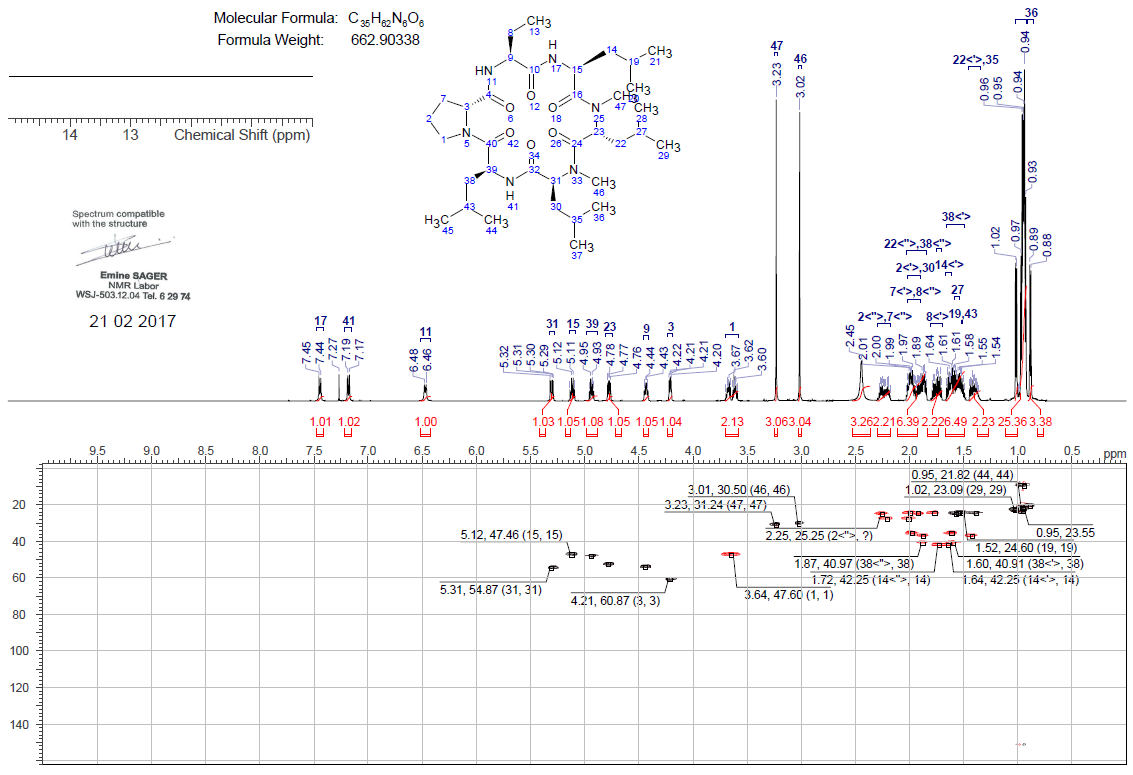 | 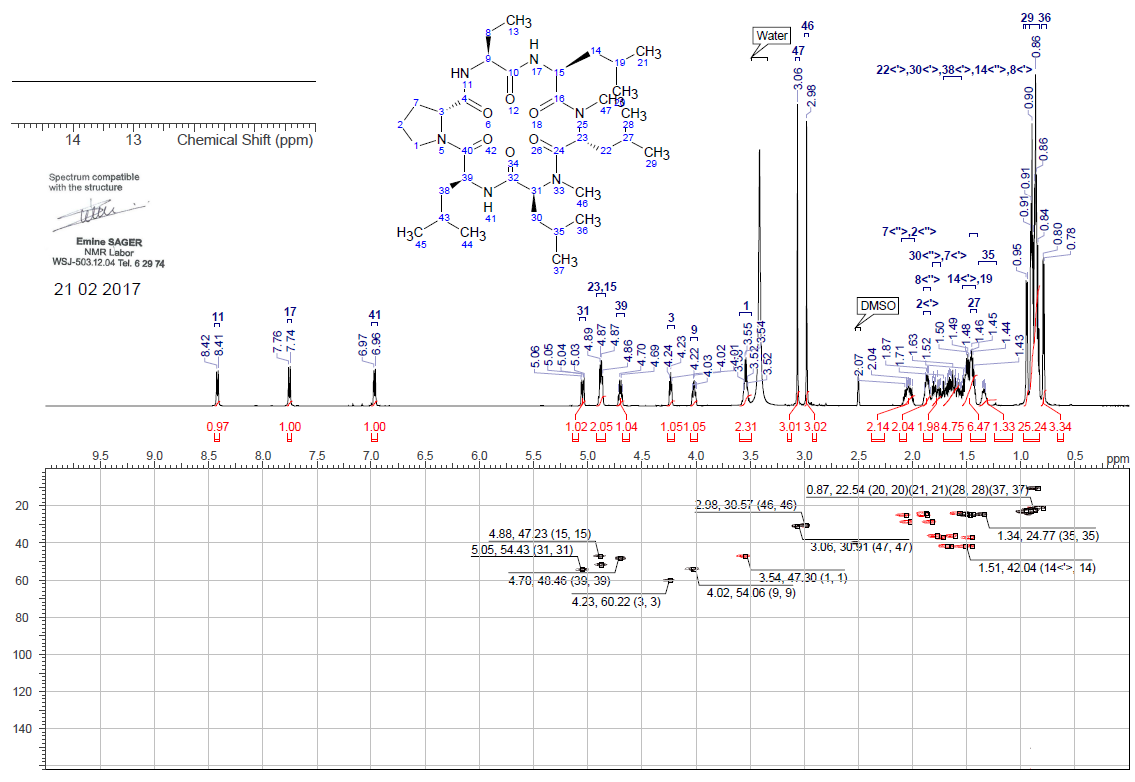 |
| Fig. S2I Compound (5) CDCl3 | Fig. S2J Compound (5) d^6^-DMSO |
| 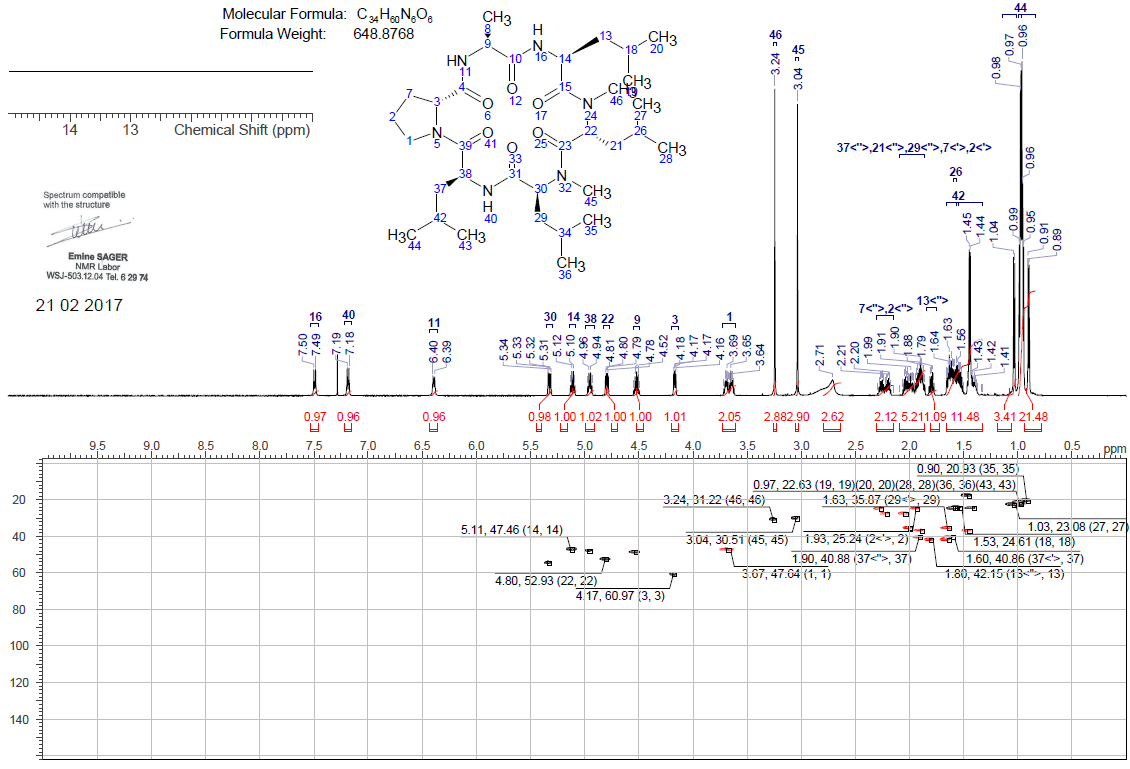 | 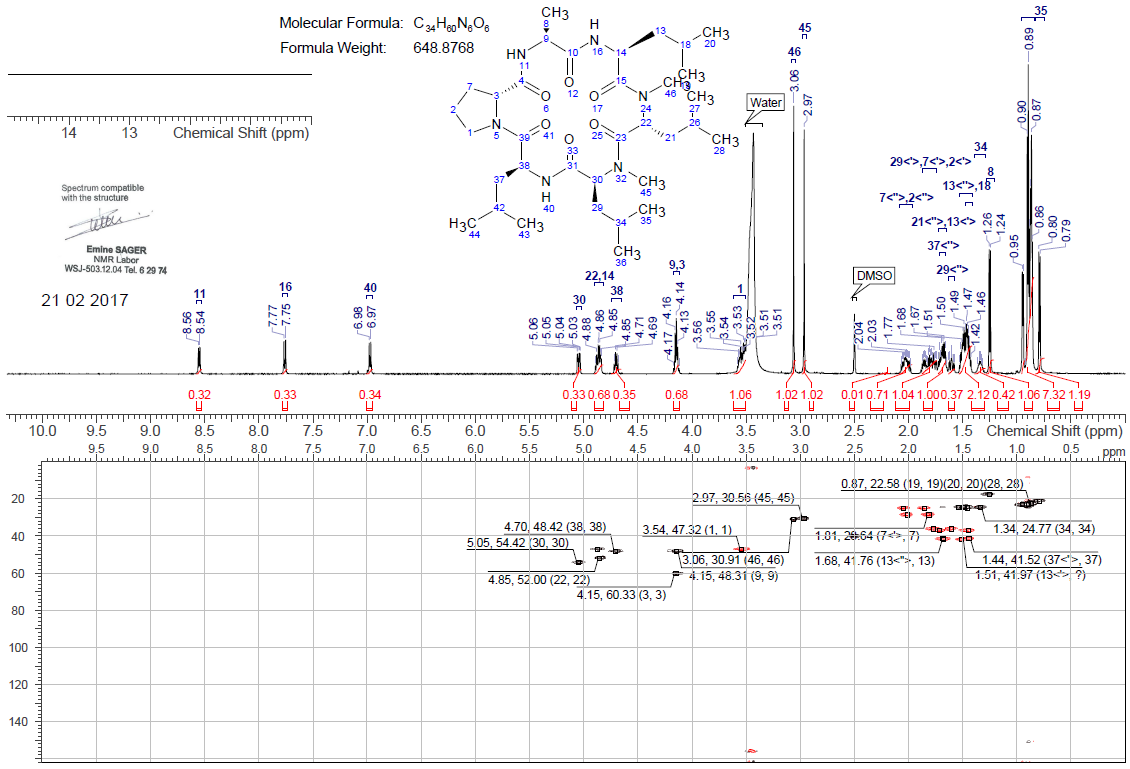 |

**Stability in kidney homogenate, liver homogenate, blood**

Determination of proteolytic stability as described in the original manuscript.

| Fig. S3A Compound (1) [Tyr-Leu-D-Leu-Leu-Leu-D-Pro-]  Stability in kidney homogenate, liver homogenate, blood: average of two determinations |
| --- |
|  |
| Fig.S3B  Compound (2) [NMe-Tyr-Leu-NMe-D-Leu-NMe-Leu-Leu-D-Pro]  Stability in kidney homogenate, liver homogenate, blood: average of two determinations |
|  |

| Fig. S3C Compound (3) [Phe-Leu-NMe-D-Leu-NMe-Leu-Leu-D-Pro]  Stability in kidney homogenate, liver homogenate, blood: average of two determinations |
| --- |
|  |
| Fig. S3D Compound (4) [Abu-Leu-NMe-D-Leu-NMe-Leu-Leu-D-Pro]  Stability in kidney homogenate, liver homogenate, blood: average of two determinations |
|  |

| Fig. S3E Compound (5) [Ala-Leu-NMe-D-Leu-NMe-Leu-Leu-D-Pro]  Stability in kidney homogenate, liver homogenate, blood: average of two determinations |
| --- |
|  |
